# Fake anabolic androgenic steroids on the black market – a systematic review and meta-analysis on qualitative and quantitative analytical results found within the literature

**DOI:** 10.1186/s12889-022-13734-4

**Published:** 2022-07-17

**Authors:** Raphael Magnolini, Luis Falcato, Alessio Cremonesi, Dominique Schori, Philip Bruggmann

**Affiliations:** 1 Arud Centre for Addiction Medicine, Schützengasse 31, 8001 Zurich, Switzerland; 2 Department of Public and Global Health, Epidemiology, Biostatistics and Prevention Institute, University of Zurich, Zurich, Switzerland; 3 Division of Clinical Chemistry and Biochemistry, University Children’s Hospital Zurich – Eleonore Foundation, Zurich, Switzerland; 4 Department of Social Welfare, Drug Information Centre ‘DIZ’, City of Zurich, Zurich, Switzerland; 5 Institute of Primary Care, University of Zurich and University Hospital Zurich, Zurich, Switzerland

**Keywords:** Anabolic androgenic steroids, Doping, Fake, Counterfeit, Substandard, Falsified, Quality, Quantity, Black market, Epidemiology

## Abstract

**Objective:**

Supraphysiologic doses of anabolic androgenic steroids (AAS) are widely used to improve body image and sport performance goals. These substances can easily be acquired over the internet, leading to a substantial black market. We reviewed literature that assessed the quality and quantity of AAS found on the black market.

**Methods:**

We searched PubMed/Medline, Embase and Google Scholar for articles published before March 2022. Additional hand searches were conducted to obtain studies not found in the primary literature search. Studies were included if they report on qualitative and/or quantitative analytical findings of AAS from the black market. Primary outcomes were proportions of counterfeit or substandard AAS. Eligible articles were extracted; quality appraisal was done using the ToxRTool for in-vitro studies. We used random-effects models to calculate the overall mean estimates for outcomes. The review protocol has been published and registered in INPLASY.

**Results:**

Overall, 19 studies, which in total comprised 5,413 anabolic samples, met the inclusion criteria, and passed the quality appraisal from two WHO world regions that reported findings, the Americas and Europe. Most studies were nonclinical laboratory studies (95%) and provided samples seized by authorities (74%). In 18 articles, proportions of counterfeit substances and in eight articles, proportions of substandard substances were presented. The overall mean estimate for counterfeit anabolic steroids found on the black market was 36% (95% CI = 29, 43). An additional 37% (95% CI = 17, 63) were of substandard quality. We also demonstrate that these drugs could contain no active ingredient, or in another amount than that labeled, a wrong active ingredient, as well as not all or more active ingredients than were labeled. High heterogeneity among all analyses and significant differences between geographical subgroups were found.

**Conclusion:**

With this systematic review and meta-analysis, we demonstrate that substantial mean proportions of black-market AAS are counterfeit and of substandard quality. These products pose a considerable individual and public health threat, and the very wide range in proportions of fake black-market AAS puts the user in a situation of unpredictable uncertainty. There is a great need for future prevention and harm-reduction programs to protect users from these substances.

**Supplementary Information:**

The online version contains supplementary material available at 10.1186/s12889-022-13734-4.

## Background

The effect of supraphysiologic doses of anabolic androgenic steroids (AAS) on muscles, especially combined with strength training, has been described and recognized in literature for decades [1–5]. AAS belong to the broader group of image and performance enhancing drugs (IPEDs) and are widely used as a convenient and easy method to improve body image and sport performance goals [6]. Global lifetime prevalence of AAS use is estimated to be as high as 3.3% within the general population [7]. Historically, the majority of AAS users were professional or competitive athletes, but nowadays survey data has revealed that over 75% of AAS users are non-competitive bodybuilders or athletes, who are mostly motivated by cosmetic benefits over performance enhancement from AAS use [4, 6, 8–13]. Due to lack of reporting, precise prevalence and demographic information on the use of these substances is challenging [10]. There are different ways to acquire illicit AAS, but the major source is described to be the internet (50–80% of acquisitions) [1, 8, 14, 15]. Injectable testosterone, synthetic AAS, other hormones and adjunctive therapies can easily be purchased over the internet and are delivered to a consumer’s home without prescription [4, 6, 8]. This provides the perfect foundation for a counterfeit drug market for all IPEDs. Isles and colleagues [16] describe the term counterfeit medicine as ‘closely associated and legally defined within intellectual property legislation and concentrates on trademark protection’, whereas they suggest the term fake medicine best serves to communicate with the public to raise awareness on this topic. The counterfeit drug market can affect all drugs and is estimated to be a multimillion dollar business [17]. These drugs may contain no active ingredient, or in another amount than that labeled, a wrong active ingredient, as well as not all or more active ingredients than were labeled. Counterfeit products can potentially lead to negative health outcomes and are considered an individual and public health threat [18]. The problem of the counterfeit market of AAS and other IPEDs and the possible dangers associated with it have already been described in 1991 [19]. Up until today there is still no effective way to protect AAS users from counterfeit AAS, as there is no formal quality control in place to ensure that what is acquired is real. Trust in the seller is described as the key criterion for protection against counterfeit drugs [20].

To further determine the proportions of fake AAS found on the black market, we conducted a systematic literature review and meta-analysis of analytical test results for those substances within the published literature. Besides the well-known side effects of anabolic steroids, new individual and public health threats arise due to fake drugs from the black market. With this systematic review we aim to further elaborate on these threats and suggest evidence-based approaches to reduce harms for this user population. To our knowledge, this is the first systematic literature review analyzing fake black-market AAS within the published literature.

## Methods

We conducted a systematic review and meta-analysis following the Preferred Reporting Items for Systematic Reviews and Meta-Analyses 2020 (PRISMA) statement [21]. The review protocol has been published previously [22] and was was registered on INPLASY (INPLASY2021110042) and is available in full on inplasy.com (https://inplasy.com/inplasy-2021-11-0042/).

### Search strategy and selection criteria

We searched PubMed/Medline, Embase and Google Scholar for studies published before March 29, 2022 that analyzed the quality and quantity of AAS to determine the proportions of substandard and counterfeit products found on the black market. We used the following search strategy with Boolean operators for PubMed/Medline and Embase: ((fake) OR (counterfeit)) AND (anabolic steroids). For Google Scholar the same search terms were used without Boolean operators. Furthermore, we continued pursuing relevant references to articles and manually tracked electronic citations related to the topic in order to identify sources in obscure locations, also called the snow-ball method [23]. The detailed search and screening strategy has been published within the review protocol [22]. Each study was screened by title and abstract based on predefined eligibility criteria (Table 1). Quality assessment for bias of analytical studies was conducted using the ToxRtool for in-vitro studies [24] and was assessed by two reviewers (RM and PB) independently. Disagreements in study eligibility, data extraction, and quality assessment were resolved by consensus between the two reviewers.

**Table 1:** Eligibility criteria

Inclusion criteria	Exclusion criteria
• peer-reviewed original articles with full-text available • no restriction regarding country and date • articles in English language or with English abstracts • articles that present proportions of original and/or counterfeit and/or substandard drugs	**•** abstract-only papers as preceding papers, conferences, editorials, and author response theses and books **•** articles without full text available **•** articles where the exact composition of analyzed IPEDs is not provided by the author **•** to increase the homogeneity, article with mixed samples (e.g., if the analysis includes different classes of IPED) in which data on AAS are < 75% of the analyzed substances

*IPED* Image and Performance Enhancing Drug(s), *AAS* Anabolic Androgenic Steroid(s)

### Classification of prohibited substances and outcomes

Classification of prohibited substances are according to the world anti-doping agency (WADA) prohibited list (Updated version as of 01 January 2021) (Table 2). We further classified compounds according to the suggested classification of Neves [25], and Weber and colleagues [26] with adaptions into “original”, “substandard” and “counterfeit”. Counterfeit means that the active ingredient does not match the label, whereas substandard means that the active ingredient matches the label, but the concentration is not as labeled. We used a subclassification of “counterfeit” substances to comprise “adulterated”, “substituted” and “inert”; and “substandard” substances to comprise “over- and under- concentrated” (Table 3). Substitution means that different active ingredients than that indicated on the label are included, whereas adulteration refers to more, or not all active ingredients that are included as indicated on the label. Primary outcomes are proportions of counterfeit and substandard substances. Secondary outcomes are proportions of adulterated, substituted, and inert substances for counterfeit results, and over-concentrated and under-concentrated substances for substandard results. Furthermore, we assessed the different analytical methods used to determine the quality and quantity of AAS on the black market.

**Table 2:** Classification of prohibited substances coded according to the WADA prohibited list (updated version as of 01 January 2021)

WADA Class	Compound Class	Examples of compounds
**S1**	Anabolic agents	E.g. anabolic androgenic steroids, other anabolic agents such as clenbuterol and selective androgen receptor modulators
**S2**	Peptide hormones, growth factors, related substances and mimetics	E.g. erythropoietins, chorionic gonadotropin, luteinizing hormone and growth hormone
**S3**	Beta-2 agonists	E.g. fenoterol, salbutamol and salmeterol
**S4**	Hormone and metabolic modulators	E.g. aromatase inhibitors (such as anastrozole, letrozole), anti-estrogenic substances (such as tamoxifen, clomiphene), myostatin inhibitors and insulins
**S5**	Diuretics and masking agents	E.g. desmopressin and acetazolamide

**Table 3:** Qualitative and quantitative analysis according to the suggested classification of Neves [25], and Weber and colleagues [26] into original, substandard or counterfeit and subclassifications with some adaptions for analysis

Classification	Description and subclassification
Original	• Formulation detected fully matches the one declared on the label/ accurately labeled (qualitative) • Levels of active pharmaceutical ingredients (AI) detected are between the defined range of the declared formulation defined by the individual study^a^ (quantitative)
Substandard	• Formulation detected fully matches the one declared/ accurately labeled (qualitative) • Levels of AI detected are not between the acceptable range defined for original products^a^ (quantitative) • Subclassification (quantitative): - Over-concentrated: AI detected above defined range - Under-concentrated: AI detected below defined range
Counterfeit^b^	• Formulation detected does not match the label/ not accurately labeled (qualitative) • Subclassification (qualitative): - Inert: no AI present - Substituted: different AI than labeled present - Adulterated: not all or more AI than the labeled AI present

*AI* Active ingredient

^a^ Adapted from Neves and colleagues’ specific range of 80–130% of the declared formulation

^b^ Adapted from Neves and colleagues: for our study there is no focus on authentic packaging, unregistered or non-existent manufacturer, lot numbers and expiry dates, or classes with no specification

### Data extraction and data analyses

Data extraction was performed independently by two reviewers (RM and LF), with disagreement resolved by discussion. The pooled proportions for primary outcomes and corresponding 95% confidence interval (CI) were calculated using a random-effect model, using the procedure for meta-analysis of single proportions “metaprop” from the library “meta”, provided in R software for statistical computing. The heterogeneity was evaluated by I^2^ statistic [27]. Publication bias was examined by funnel plots [28, 29]. A subgroup analysis was conducted for counterfeit AAS (proportions of adulterated, substituted and inert substances), substandard AAS (proportions of over-concentrated and under-concentrated substances) and based on geographical location. The detailed data extraction and data analysis plan have been published elsewhere [22]. Meta-regression analyses provided in R software were conducted to explore the association between studies’ publication year and outcome measures [30].

## Results

### Selection of eligible studies

**Fig. 1:**
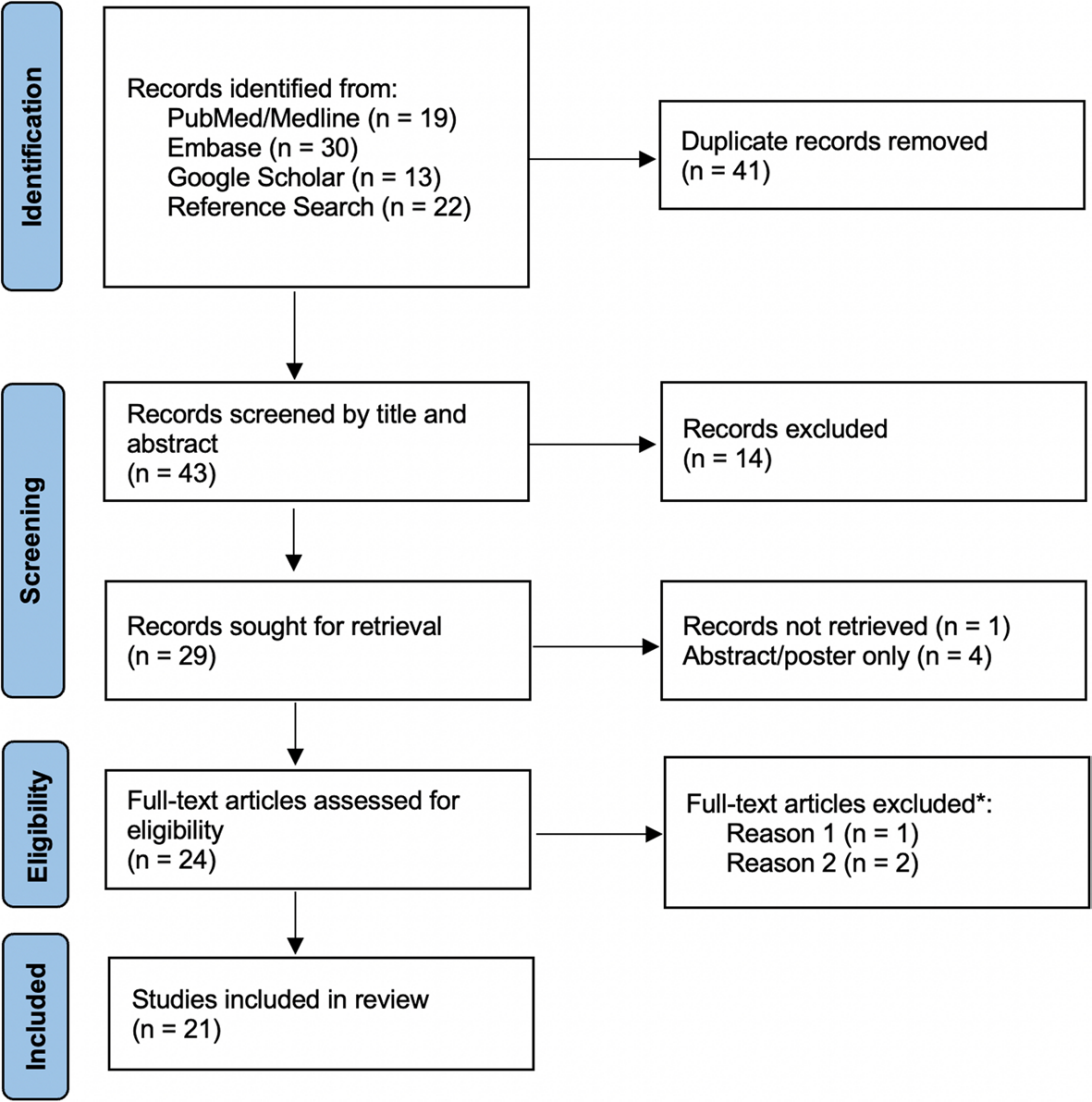
PRISMA flow diagram. Reasons for exclusion of full-text articles: *Reason 1: Qualitative and quantitative analysis for products not labeled for AAS were conducted [31]; Reason 2: No qualitative or quantitative laboratory analysis of seized compounds was done [32, 33].

The flow diagram of literature searches and results is shown in Fig. 1. With the defined search strategy, we identified a total of 84 records (PubMed/Medline: 19 (31 hits); Embase: 30 (63 hits); Google Scholar: 13 (487 hits); reference search: 22) that led to a total of 43 titles and abstracts that were screened after the removal of duplicates. We retrieved a total of 24 full-text articles from these different sources. One record was not obtained in full-text format and four records were abstracts or posters only and were therefore excluded. The full-text screening stage of 24 articles led to 21 potential articles relevant in this systematic review and were thus eligible for quality appraisal. Additional articles were excluded after full-text assessment for the reasons mentioned in the flowchart (Fig. 1).

### Quality appraisal of the included studies

A total of 20 full-text articles were included for quality appraisal by the ToxRTool. All quality appraisal results can be found in Supplementary file 1. Most studies (*n* = 18) analyzed by the ToxRtool reporting quantitative and qualitative data were appraised with strong ratings and high reliability scores (reliable without restrictions, reliability category 1). The minority (*n* = 2, [15, 20]) scored weak ratings and low reliability scores (not reliable, reliability category 3) as they did not provide enough information on their test system characterization or study design description and were therefore excluded. For one study, the study design (retrospective database analysis) did not qualify for the analysis by ToxRtool and was individually assessed by the study team [34]. The authors provided sufficient information in the methods section so that, by consensus between the reviewers (RM/PB/LF), we were confident to include the study for extraction and analysis. After the quality appraisal stage, an overall number of 19 full-text articles were included for data extraction and analysis.

### Study characteristics

All study characteristics can be viewed in detail in Table 4. The peer-reviewed literature of qualitative and quantitative analyses of AAS has considerably increased in the last few years. Among the included studies, the majority (53%) were published in the last five years of this current study (2017–2022) and the vast majority (79%) were published within the last decade (2012–2022) of this current study. The mean year of publication was 2017.

**Table 4:** Characteristics of 19 published studies presenting qualitative and quantitative data of fake AAS on the black-market

**Characteristic**	
Year of publication (mean)	2017 (1997 to 2021)
• Published within 5 years	• 10 (53%)
• Published within 10 years	• 15 (79%)
Sample information	
• No. of samples included (mean; median)	• 285; 42.0
• Range of samples included (min; max)	• 8; 2818
• Cumulative sample size	• 5,413
Study design	
• Retrospective database analysis	• 1 (5%)
• Nonclinical laboratory studies	• 18 (95%)
No. of included studies presenting	
• Anabolic agents (S1)	• 17 (89%)
• Mixed samples	• 2 (11%)
No. of WHO regions and countries included	
WHO Region of the Americas (AMR)	7 (37%)
• Brazil	• 7 (100%)
European Region (EUR)	12 (63%)
• Switzerland	• 1 (8.3%)
• France	• 1 (8.3%)
• Italy	• 2 (16.7%)
• Germany	• 3 (25%)
• United Kingdom	• 1 (8.3%)
• Czech Republic/Slovakia	• 2 (16.7%)
• Belgium	• 1 (8.3%)
• Austria	• 1 (8.3%)
Sample collection methods	
• Seized compounds by authorities	• 14 (74%)
• Bought directly from the black market	• 4 (21%)
• Received directly from gyms and users	• 1 (5%)
Articles presenting outcomes	
Counterfeit substances	18 (95%)
• Inert substances	• 10 (56%)
• Substituted substances	• 10 (56%)
• Adulterated substances	• 9 (50%)
Substandard substances	8 (41%)
• Over-concentrated	• 4 (50%)
• Under-concentrated	• 4 (50%)
Original substances	
• Qualitative analysis only	• 18 (95%)
• Qualitative and quantitative analysis	• 7 (37%)

The geographic scope of the included studies is limited to two world regions, where 37% and 63% respectively were conducted, and these studies reported findings from the Americas (AMR) and Europe (EUR). Research in the Americas was only done in Brazil, which alone includes 7 of the 19 studies. In the case of Europe, the studies are divided among several countries. The country with the highest number of included studies in this region is Germany with a total of three studies. In addition, other countries from this region (Switzerland, France, Italy, United Kingdom, Czech Republic and Slovakia, Austria, and Belgium) are represented in our list of included studies. The studies included a median of 42 samples; the largest study had 2818 analyzed samples and the smallest 8 samples, and a cumulative sample size of 5,413 anabolic agents.

Most included study designs (95%) were nonclinical laboratory studies. One study was a retrospective database analysis of the Brazilian federal police database [34]. Most samples (74%) originated from seized compounds by the police, custom authorities, or justice departments and a minority of samples were bought directly from the black market or provided by gyms and users themselves.

In 17 articles we were able to extract samples that exclusively analyzed anabolic agents (WADA class S1). Some articles also included other classes of substances in their analysis, such as WADA classes S2, S3, S4, S5, dietary supplements, stimulants, and sexual performance enhancers. Importantly, whenever anabolic agents were analyzed with other classes of substances, anabolic agents made the highest proportion of analyzed classes. In two articles, the authors analyzed mixed samples, but the proportion of AAS was above 75%, as described in the inclusion criteria published in the study protocol [34–36].

### Data extraction

The full extraction form can be found in Supplementary file 2; the summary form used for data analysis can be found in Supplementary file 3. In seven articles (37%), both main endpoints were presented simultaneously. In 18 articles, counterfeit substances and only in eight articles, substandard substances were presented. For counterfeit substances, most studies sub-analyzed data into inert, substituted, and adulterated samples. Half of the studies presenting data on substandard substances were sub-analyzed into over-concentrated and under-concentrated samples. For most original substances, we were able to extract qualitatively analyzed data (accurately labeled) and only for 37% were we able to extract qualitatively and quantitatively analyzed data (accurately labeled and concentration within range as declared on the label).

### Data synthesis of fake AAS found on the black-market

#### Counterfeit anabolic steroids

**Fig. 2:**
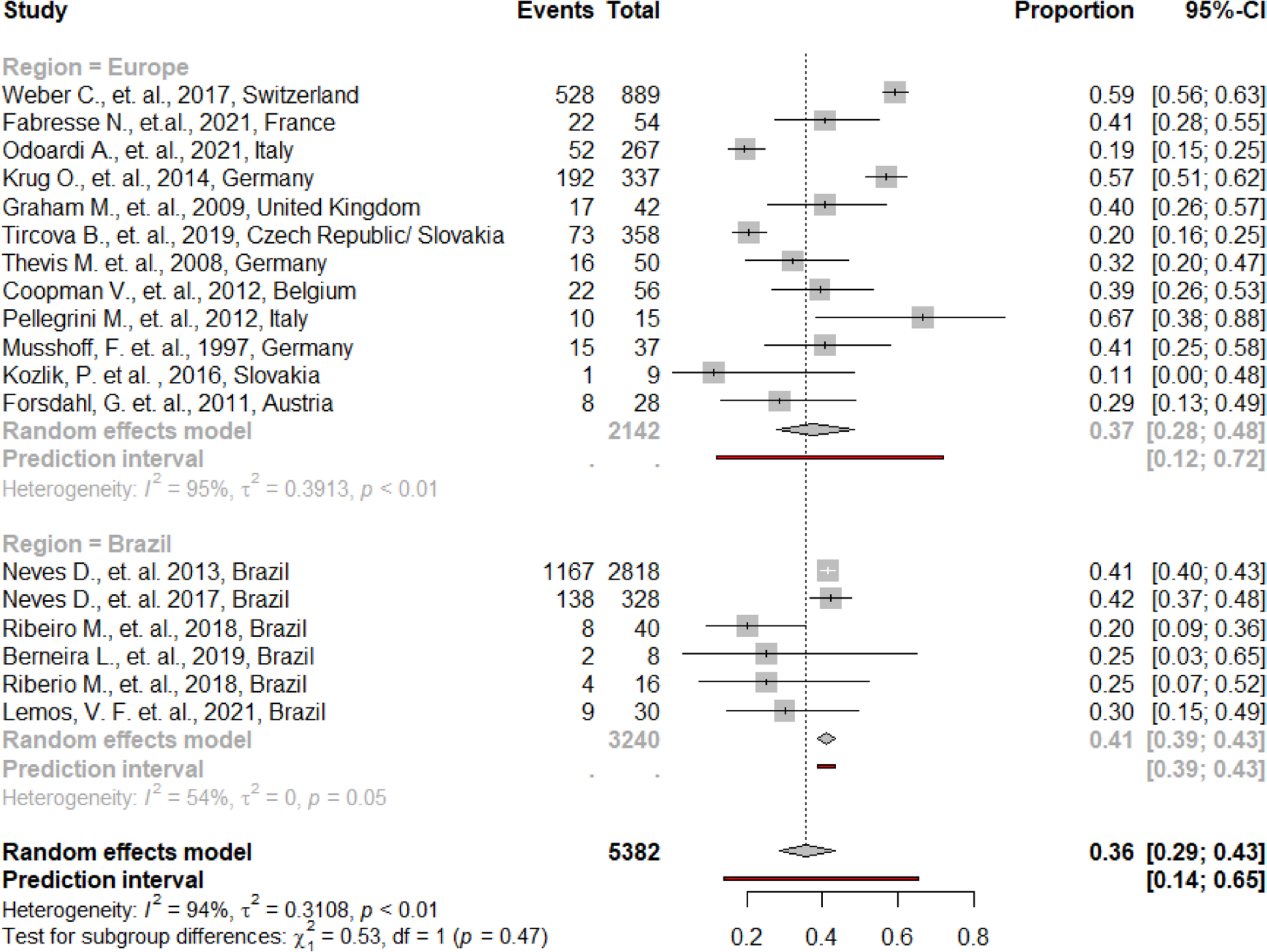
Proportions of counterfeit anabolic androgenic steroids from 18 studies, grouped by geographical region

The overall mean estimate for counterfeit AAS was 36% (95% CI = 29, 43), with prediction intervals ranging from 12 to 72% in European countries, and from 39 to 43% in Brazil. High heterogeneity was demonstrated (I^2^ = 94%, *p* < 0.01), but no significant difference (*p* = 0.47) between the two geographical regions was found (Fig. 2). All main analyses are provided in Supplementary file 4.

Sub-analyses for counterfeit anabolic agents demonstrate that those substances can be inert, substituted or adulterated, with overall mean estimates of 24% (95 CI = 9, 49), 44% (95 CI = 27, 63) and 11% (95 CI = 2, 42), respectively. High heterogeneity was demonstrated in all sub-analyses. Interestingly, significant differences (*p* < 0.05) for the two geographical regions were found. The mean estimate for inert substances was significantly higher in Brazil (49% vs. 15%), whereas estimates for substitution of AAS were significantly higher in Europe (51% vs. 28%). No significant difference (*p* = 0.47) was found for adulteration between the two regions. All sub-analyses are provided in Supplementary file 5.

Substitution of AAS could occur with i) AAS of the same steroid class (e.g. different testosterone esters (testosterone enanthate or propionate instead of testosterone isocaproate [26]; ii) AAS of different steroid classes (e.g. in parental preparations: testosterone or trenbolone esters instead of nandrolone, drostanolone or methenolone esters; in oral preparations: stanozolol instead of oxandrolone [26]); iii) completely different compound classes according to the WADA prohibited list (e.g. anastrozole (aromatase inhibitor) instead of mesterolone [37]); or iv) completely different pharmaceuticals (e.g. quinine (antimalarial drug) instead of methandienone [37]). Examples of adulterated samples were found where not all active ingredients were included as indicated on the label (e.g. Testomix 300 only included testosterone propionate instead of a mixture of testosterone esters (testosterone propionate, phenylpropionate, isocaproate and decanoate [37]), or additional active ingredients were included than those indicated on the label (e.g. Boldenone 200 mg (boldenone undecylenate) additionally included testosterone propionate [37]).

#### Substandard anabolic steroids

**Fig. 3:**
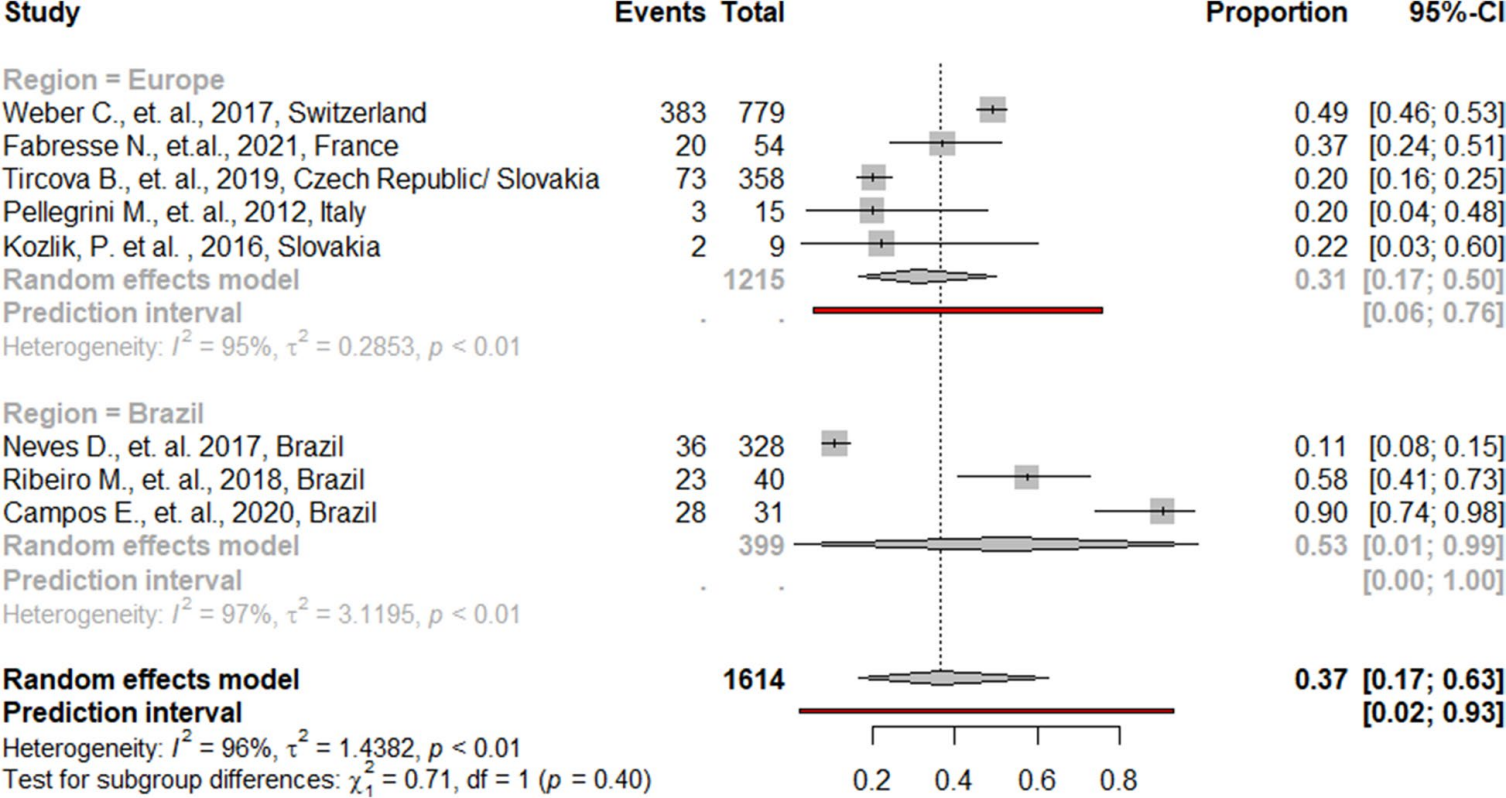
Proportions of substandard anabolic androgenic steroids from 8 studies, grouped by geographical region

The overall mean estimate for substandard AAS was 37% (95% CI = 17, 63), with prediction intervals ranging from 6 to 76% in European countries, and from 0 to 100% in Brazil. High heterogeneity was demonstrated (I^2^ = 96%, *p* < 0.01), but no significant difference (*p* = 0.40) between the two geographical regions was found (Fig. 3). All main analyses are provided in Supplementary file 4. Sub-analyses for substandard AAS demonstrated that these substances appear to be more under-concentrated than over-concentrated, with overall mean estimates of 67% (95 CI = 19, 94), compared to 33% (95 CI = 6, 81) respectively. High heterogeneity was demonstrated in both sub-analyses. Significant differences (*p* < 0.01) for the two geographical regions were found. The mean estimate for over-concentrated AAS was significantly lower in Europe compared to Brazil (12% vs. 64%). All sub-analyses are provided in Supplementary file 5. Some authors (not included in analysis) declared that most, or even all of the tested AAS were concentrated below what was stated on the label, without further quantification of the analyte(s), providing more evidence that AAS are more likely to be under-concentrated than over-concentrated [37–41].

The analysis for substandard substances comes with some challenges. Firstly, defined ranges of declared labels could vary massively between articles, had a quantitative analysis been performed, with defined ranges between 50–200% [26], 80–130% [42], 80–120% [43] or 90–110% [44]. In some studies, the contained active ingredients in “under-concentrated” preparations was much lower than 50% of that indicated (e.g. 0.5–1.5% [45], 9% [44] or 16% [46]) if quantitative data was available. For “over-concentrated” preparations however, active ingredients could go as much as 200% above that indicated on the label (e.g. 221% [25] or 225% [44]) if quantitative data was available. Furthermore, most authors (*n* = 7) performed a quantification only in the accurately labeled substances, whereas Weber and colleagues [26] included mixed samples (accurately labeled and adulterated) for quantitative analysis.

**Fig. 4:**
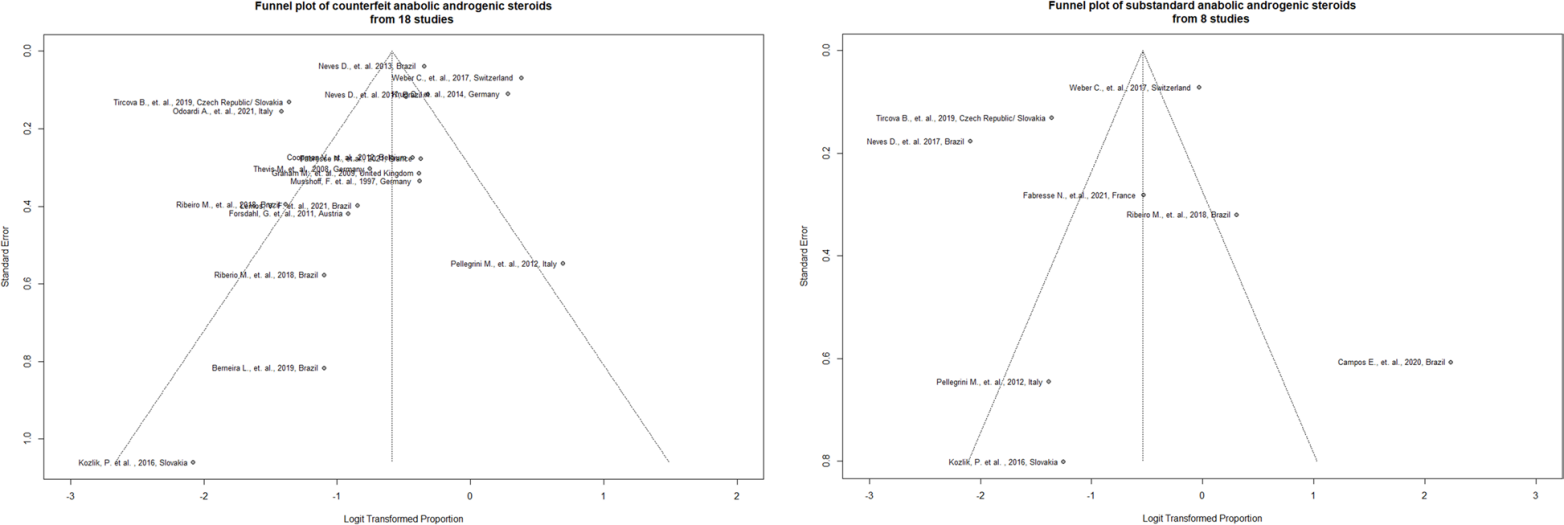
Funnel plot for counterfeit AAS (left), funnel plot for substandard AAS (right). Note that the “desirable result” (low proportion) is on the left side of the plots

Funnel plots (Fig. 4) show the plots of the logit transformed proportions from each study (x-axis) against its standard error (y-axis) for counterfeit and substandard AAS, as a measure of precision of that study. If smaller, statistically not significant studies tend to remain unpublished, then an asymmetrical shape may be observed. However, any factor which is associated with both study outcome and study size could confound the true association and cause asymmetry [29]. Both the visual evaluation of the plots as well as the non-significant results (counterfeit: *p* = 0.44; substandard: *p* = 0.98) of Peters’ linear regression test of funnel plot asymmetry [28] do not point to such biases. Yet as the plots do not show a funnel shape in our meta-analysis, the studies’ sample size is not associated with the study outcome. This is in line with the fact that we cannot expect a “true proportion” in reality. Rather than caused by study design issues, the differing proportions of counterfeit or substandard AAS reflect the selection of the tested AAS samples, with real differences in the quality of AAS found on the black market. Meta-regressions showed that the studies’ publication year did not influence the found proportion of counterfeit (β =—0.03, *p* = 0.23) and substandard AAS samples (β = 0.23, *p* = 0.21). All main analyses are provided in Supplementary file 4.

#### Additional outcomes and findings

Some authors have analyzed and compared the quantity and quality of different AAS formulations. Both the proportion of substandard and counterfeit products are described to be higher in formulations for oil-based solutions used for injectables compared to tablets used for oral administration [25, 26, 36, 43]. Counterfeit proportions for oil-based solutions compared to tablets are described as 43–65% vs. 29–37%, respectively [25, 43].

Furthermore, Graham and colleagues [36] further analyzed injectables for intramuscular injection for sterility. Microbiological cultures of samples revealed the presence of contaminants that were identified as bacterial skin commensals.

Some authors assessed visual inspection of packaging and detection of counterfeiting rate with contradicting results. Thevis and colleagues [47] demonstrated that visual inspection did not allow a differentiation between original and counterfeit products, whereas Berneira and colleagues [40] demonstrated that visual inspection supported instrumental characterization of AAS and that it was a crucial procedure in order to characterize and detect falsifications.

Samples commonly originated from many different countries and manufacturers [26, 43]. As an example, Weber and colleagues [26] analyzed 1,190 seized IPEDs at the Swiss border and identified 204 different manufacturers and 48 countries of origin, predominately manufactured in Asian countries, that were sent to Switzerland mostly via South Eastern European countries [48]. Tircova and colleagues [43] analyzed 358 voluntarily obtained anabolic steroids and identified 49 different manufacturers, the majority of them being underground labs and only the minority being pharmaceutical companies. Neves and colleagues [34] described that the majority of seized substances in Brazil (*n* = 3,537) originated from Paraguay and Brazil itself, whereas a minority originated from outside Latin America.

There is some evidence within the analyzed literature that the amount of seized or confiscated compounds increased over the observation period [26, 34], with one documented significant, i.e. 5.2-fold increase of seized anabolic steroids (1,468/282) over a 5 year period [34].

### Analytical techniques used for quantitative and qualitative analysis of anabolic steroids

Over the past years, different analytical techniques have been used to screen, identify, and quantify AAS. Among the included studies, most approaches are based on liquid chromatography coupled to mass spectrometry (LC–MS/MS) [32, 42, 47, 49, 50], or gas chromatography coupled to mass spectrometry (GC–MS) [32, 35, 40, 42, 47, 49, 51–54]. GC–MS with [51] or without [40, 46] prior derivatization of the AAS has also been successfully used to screen and quantify AAS, based on their fragmentation patterns and retention times. Other complementary techniques like 1H-nuclear magnetic resonance (NMR) [39, 44], infrared (IR) spectroscopy [34, 40, 46, 55], differential scanning calorimetry (DSC) [40], or high resolution/high accuracy mass spectrometry (LC-HRMS) [26, 32, 42] have also been used to measure AAS. For AAS, both low-resolution and high-resolution mass spectrometers were employed. The sample preparation for LC–MS/MS or GC–MS is simple and was mostly based on an extraction with organic solvents, usually methanol [43, 46, 47, 49, 51], followed by sonication. Oil-based preparations were directly extracted with the appropriate solvent, while tablets and capsules were grounded into a fine powder before extraction.

## Discussion

### Quality and quantity of anabolic androgenic steroids found on the black market

In this systematic review, we were able to include 19 articles within the published literature that provided qualitative and/or quantitative analytical test results of AAS found on the black market from 9 different countries (eight in Europe; one in Latin America), with a cumulative sample size of 5,382 products being analyzed qualitatively and 1,614 being quantitatively tested. We demonstrate that substantial proportions of AAS found on the black market are fake. The overall mean estimate for counterfeit anabolic steroids found on the black market was 36% (95% CI = 29, 43), and an additional 37% (95% CI = 17, 63) were of substandard quality. Although these proportions must be interpreted with caution due to some methodological challenges and high heterogeneity, one must acknowledge the unreliable nature of those substances acquired from the black market. The very wide range in the proportions of counterfeit or substandard AAS from the black market shows the uncertainty about quality, thus leaving users with unpredictable risks. AAS were the most dominant group within all analyzed products, and they were almost exclusively analyzed within the WADA class S1. We demonstrate that fake AAS can be substituted, not contain any substance at all, or be adulterated. But in addition, products that contain the labeled substances can still be over-concentrated or under-concentrated. Interestingly, this systematic review showed significant differences between the two included world regions. In Europe, AAS from the black market appear to be more likely to be substituted and less likely to be inert, but also less likely to be over-concentrated compared to Brazil. Substandard and counterfeit products found in our systematic review were most likely produced by manufacturers not in line with good manufacturing practices (GMP’s) [56]. Rather, those products are produced in clandestine underground laboratories lacking the necessary knowledge or equipment to produce these compounds in adequate quantity and quality, as also described by other authors [26, 35, 38]. The shift from pharmacies to deregulated underground online sites and clandestine underground laboratories occurred after the United States enacted the Anabolic Steroid Control Act in the 1990s. Underground laboratories emerged both locally and in countries with lax legal regulations and it is described that an 'anabolic steroid tourism' and large networks of online resellers emerged, simplifying the illegal acquisition of anabolic steroids [57].

Different reasons may be responsible for the discrepancies between the declared label and actual content demonstrated in our systematic review, such as i) intentionally removing or exchanging expensive AAS with cheaper ones, or diluting AAS in order to increase the manufacturers’ profit; ii) unintentionally, due to contamination and inadequate decontamination of machines that are used for the production of different active ingredients; iii) poor quality of production where possible heterogeneity within the same production batch occurs due to inadequate mixing of active ingredients and diluents; iv) inadequate post-production, where packages and labels are switched; and v) inadequate shipment and storage conditions where changes in the active ingredient and diluents could occur, or even shipment of expired pharmaceuticals [6, 26, 38]. We provide further evidence that the amount of seized or confiscated compounds increased over the observation period [26, 34], up to 5.2-fold in a 5 year period [34]. This is in line with current trends observed in AAS user surveys that the popularity of AAS has significantly increased over the past decade [6].

We demonstrate that visual inspection of the package, label, and internal content to identify preliminary signs of counterfeiting of AAS have shown to be mostly ineffective. Although these methods may be useful for some suspected samples, this must be further supported by analytical techniques. There is a broad availability of different analytical tools used to identify counterfeit AAS on the black market, as included in this systematic review. Although approaches using gas and liquid chromatography coupled to mass spectrometry as well as spectroscopic techniques were most frequently used for this systematic review, novel techniques have been developed in the recent past. Analytical methods can vary considerably in terms of instrumentation cost, analysis time, and identification and quantification software. Most analytical approaches require sophisticated instruments that need considerable budget and skilled personnel to operate them, which might limit their use in certain settings. The broad diversity of different techniques that were applied may also lead to substantial heterogeneity within our analyses.

We further show a limited geographical scope of included studies, with all studies being from countries in Europe or Brazil. Surprisingly, we did not identify any studies from the US, Middle East, Oceania, Asia, or Africa. We hypothesize different reasons, such as the paucity of studies on AAS use and major differences in prevalence of AAS use in many of the world regions mentioned above [7], sensitization for and awareness of fake drugs from unregulated drug markets through services such as ‘drug checking services’ or ‘needle exchange programs’ which are widely accessible in European settings [58], but also the wide range of global drug policies and punitive laws which are less strict in Europe compared to other countries.

### Individual and public health impact of fake anabolic agents

With this systematic review and meta-analysis, we present significant findings for fake AAS on the black market. The implication of our findings on individual and public health may be substantial and we want to highlight the following threats:

#### Compound-specific adverse events and side effects

Different anabolic steroids come with compound or class-specific and unspecific adverse events. Fake products can lead to unexpected adverse events in addition to the already well-established side effects of AAS, which can include cardiovascular toxicity, cardiotoxicity and arrhythmia, cardiovascular events (stroke, coagulation), genitourinary and reproductive impairment, sexual dysfunction and testicular atrophy, gynecomastia, central nervous system abnormalities, impaired mental health and behavior including suicide, skeletal-muscular pathologies, metabolic decompensation, impaired liver functions, and even death [1, 4, 6, 18, 59, 60]. Importantly, there are more than 60 different anabolic androgenic steroids listed on the WADA prohibited list and novel compounds are frequently detected on the market. We want to highlight one particular adverse event of those substances that can become a motivator for continued use and an increased risk of continuously being exposed to counterfeit or substandard substances, the “AAS dependence syndrome” [6]. Literature suggests that 25–40% of AAS users demonstrate AAS dependence [6, 8, 9, 14]. It is described as continuous or chronic AAS use, despite prominent adverse medical, psychological, or social effects [6].

#### Formulation and application

AAS are administered in different ways, including oral, injectables (water or oil-based), transdermal (cream or gel), buccal and sublingual [1]. The most common route of administration is per intramuscular injection [10] and we demonstrate that proportions of counterfeit and substandard substances for injectables compared to oral formulations may be considerably higher. Different forms of formulations and administrations additionally come with specific adverse events. As an example, 17α-alkylation of steroids which is used for oral administration is described to result in increased liver toxicity compared to injectable AAS, because of first-pass metabolism and increased duration time in the liver due to slow metabolization [1]. Different non-scientific and anecdotal patterns and duration of use are described in literature with the goal of minimizing side effects or maximizing the drug effects of AAS [1, 15]. Unknowingly taking the wrong formulation can lead to unexpected side effects, especially when taken over a longer period than intended or in combination with other substances.

#### Mislabeling

In this systematic review we demonstrate that the real composition, the type of production, concentration, quantity, quality, and purity are often not declared on the label, and labels are even misleading. In the case of mislabeled AAS acquired on the black market, it is currently not exactly known what is consumed by the user. We provide evidence that AAS are more likely to be under-concentrated than over-concentrated if they are of substandard quality. Anabolic steroid users commonly exceed 10 to 100 times the physiological limits [1]. This is amplified by unintentional intake of over-concentrated AAS, which can come with several severe health risks. Under-concentration can also lead to possible risks, as results on performance and image do not occur and that may lead to a much higher intake of amounts by the user. We demonstrate that on some occasions completely different pharmaceuticals were identified during the analysis, such as quinine (antimalarial drug), instead of AAS. This can, taken unknowingly, lead to substantial drug-related side effects.

#### Sterility issues

Besides the problems with chemical quality, our systematic review provides further evidence of microbiological contamination of those substances. Products from clandestine laboratories do not go through microbiological quality control, which can lead to sterility issues and microbiological contamination of injectables. Graham and colleagues [36] demonstrated contamination with bacterial skin commensals during microbiological analysis of their samples. This is especially concerning when those substances are injected into the muscle as it poses a risk of forming abscesses in the muscle and skin necrosis [36, 61].

#### Polypharmacy

Athletes are inclined to polydrug use. Drugs are used for reducing side effects of AAS abuse and/or boosting AAS effects. In addition, recreational drugs are also commonly consumed. The polypharmacy behavior with concurrent use of different licit and illicit, and possibly counterfeit substances may contribute to the toxicity of AAS, and may lead to additional unintended drug-drug interactions, also making it difficult to confirm the causal relationship between a specific substance and its adverse effect [1, 6, 10].

There is a large and increasing number of individuals who are possibly exposed to these fake AAS on the black market. We demonstrate that foreign shipments of fake AAS over the past years may have increased significantly, thus the negative consequences on public health may be substantial. In 2014, Sagoe and colleagues [7] estimated, in a systematic review, that the global lifetime prevalence of AAS use was as high as 3.3% in the general population, but may be as high as 6.4% for males and 1.6% for females [7]. Updated numbers are urgently needed, as the popularity of these substances is described to have increased, i.e. in the UK it is estimated that AAS popularity has doubled within the 10 years to 2018 [6]. Furthermore, first time use of anabolic agents has already been described in high-school age adolescents [15]. Lifetime prevalence of AAS users in recreational sportspeople and athletes is estimated to be significantly higher than the general population, with estimates of 18.4% and 13.4%, respectively [7]. Furthermore, due to punitive laws, stigma, and inexperience of health care professionals, this user population is widely unaccessed. Information about the use of AAS is commonly acquired from non-medical sources, such as word of mouth propaganda from athletes, dealers and bodybuilders [15], as there is major distrust and lack of confidence by AAS users towards medical doctors [4, 9, 14]. Therefore, it is of great importance that clinicians, politicians and law-makers are aware of this considerable individual and public health threat, given the significant negative long-term health impact of AAS misuse and exposure to fake AAS. Although striving for abstinence of those substances is the preferred way, this strategy has proven to be inefficient over the past decades, even more leading to a massive unregulated black market for doping agents. Effective harm reduction strategies, evidence-based guidelines and interventions are urgently needed to protect users from counterfeit products found on the black market, and to support the development of effective services.

### Harm reduction strategies for fake anabolic steroids and steroid users

Different harm reduction strategies could be employed to limit this user community from either getting in contact with fake AAS from the black market or to promote safer use and informed decision making. One strategy could entail the controlled use and availability of these substances through proper health channels. This strategy has already been employed in other fields of addiction medicine already. In the opioid field, one of the most effective harm reduction measures is the medical prescription of opioids (opioid agonist therapy), together with psychosocial, interdisciplinary care [62]. Such a measure is also conceivable for the AAS sector and should be further evaluated. Another strategy could entail the introduction of specialized drug checking services for this user community. ‘Drug checking’ allows people who consume illegal and legal drugs acquired from unregulated drug markets to submit samples for chemical analysis and receive feedback on the quantity, quality, and purity of those substances. Commonly this approach is embedded in a wider prevention approach that includes counselling services and other short interventions [63, 64]. Drug testing services can be an effective harm reduction service that may strengthen surveillance of the black-market drugs that are used and can accurately inform users about the quality and quantity of black market AAS prior to use [65].

Recreational drug testing services became available in the 1990s [66]. Ever since, recreational drug testing is being conducted in a growing number of countries. By 2017, a global review of drug checking services estimated that approximately 31 different drug checking programs across 20 geographical locations existed, predominantly across European countries [58], with recent expansions in Canada and the United States as a response to the emerging global opioid crisis [65–71].

There is some evidence in literature of the positive impact of drug testing services on all levels of prevention, such as accessing user populations, facilitating social support, increasing knowledge among users, improving drug-taking behavior and safer use, increasing risk awareness of recreational drug users and postponing the onset of first use, while not increasing or encouraging consumption, or extending the circle of users [64, 67–69, 72]. It is described that in order to provide good drug testing services, there must be a close collaboration between different stakeholders and actors, such as politicians, the police and medical treatment services [67].

To our knowledge, there is currently no published evidence on the controlled use of prescription testosterone through health care channels or drug testing services for AAS and other IPEDs yet. There is a considerable need for programs addressing harm reduction, prevention, and treatment among the AAS user community. Those who report using AAS and other IPEDs for non-medical purposes are aware of the problem with counterfeit drugs and possible health consequences associated with it, and take steps to limit coming into contact with these products [73]. Unfortunately, there are currently major limitations in identifying these fraudulent products by users and such services may become a cornerstone in accessing this hard-to-access user population [73]. These harm reduction interventions may be especially effective in this user population as these drugs are used for an average of 20 weeks [6], compared to recreational illicit drug use that is often only used sporadically.

### Limitations

This research is subject to some limitations. The evidence base for this research area is very limited and there are many variables in this study that may lead to the extensive heterogeneity observed within analysis, i.e. multiple data sources, small sample sizes, inconsistent study designs, diverse sample acquisition methods (seized samples vs. provided samples), different sample preparation and sample analysis methods, different sample origins and manufacturers (e.g. Asia for Switzerland vs. Latin America for Brazil), and heterogenous sample formulations (i.e., tablet/capsules for oral use vs. water- or oil-based injectables for intramuscular use). This may have resulted in some bias of the studies included and a bias in our statistical summary and conclusions.

Another limitation of this systematic literature review is the complete reliance on previously published research and the availability of these studies using the methods outlined in the search methodology and the appropriateness of these studies to the criteria of the selection/exclusion procedure. In some cases, the published data had to be manually adapted and transferred to fit our classification system. This also may have led either to over or under estimation of certain proportions of the estimates of “substandard” or “counterfeit” anabolic androgenic steroids and their subclassifications and sub-analyses.

## Conclusion

With this systematic review and meta-analysis on black-market AAS, we have demonstrated that substantial mean proportions may be of substandard quality or counterfeit. The very wide range in proportions of counterfeit or substandard black market AAS puts the user in a situation of unpredictable uncertainty. We further elaborated and highlighted reasons for the vast amount of substandard and counterfeit AAS, the individual and public health impact of those mislabeled products and the possible positive impact of harm reduction strategies for this user population. There is a great need for future prevention, harm reduction, and treatment programs for this growing and hard to reach user community.

## Supplementary Information

**Additional file 1.** ToxRTool quality appraisal results.

**Additional file 2.** Extraction form.

**Additional file 3.** Summary extraction form used for data analysis.

**Additional file 4.** Main outcome analyses.

**Additional file 5.** Supplementary analyses.

## Data Availability

All data generated or analyzed during this study is included in this published article and its supplementary information files.

## Abbreviations

AAS: Anabolic Androgenic Steroid(s)
AI: Aromatase Inhibitors
DSC: Differential Scanning Calorimetry
GC–MS: Gas Chromatography coupled to Mass Spectrometry
GMP: Good Manufacturing Practices
HRMS: High-Resolution Mass Spectrometers
IPED: Image and Performance Enhancing Drug
IR/UV: Infrared/Ultraviolet
LC-HRMS: Liquid Chromatography coupled to High Resolution Mass Spectrometry
LC–MS: Liquid Chromatography coupled to Mass Spectrometry
MRM: Multiple Reaction-Monitoring
NMR: Nuclear Magnetic Resonance
SERM: Selective Estrogen Receptor Modulator
UK: United Kingdom
WADA: World Anti-Doping Agency
μATR-FTIR: Attenuated Total Reflection Fourier Transform Infrared Microspectroscopy
