# Supplementary material for: Fake anabolic androgenic steroids on the black market – a systematic review and meta-analysis on qualitative and quantitative analytical results found within the literature
Source: BMC Public Health. 2022 Jul 17;22:1371. doi: 10.1186/s12889-022-13734-4 (PMC9288681; doi:10.1186/s12889-022-13734-4)
Supplement: Supplementary file 2 — Additional file 2. Extraction form. [file 12889_2022_13734_MOESM2_ESM.pdf]

Supplementary file 2: Extraction form

Document entitled: Fake Anabolic Androgenic Steroids on the Black Market – a systematic review and meta-analysis on qualitative and quantitative analytical results found within the literature

Author/Year/ Country/Ref	Study period	Analytical methods	Sample Information	Classes of compounds/ Sample size Number	Prop. of originals	Prop. of substandards	Prop. of counterfeit	Remarks
Weber C., et. al., 2017, Switzerland [1]	2013-2014	LC-MS/MS, SDS-PAGE coupled with nano LC-HRMS, HCG Immunoassay	Seized by Swiss customs authorities	S1, S2, S4<br>Total N=1190<br><br>S1: N = 889 (75%)<br><br>S2: N = 146 (12%)<br>S4: N = 113 (9%)	40.6%* (361/889)	49.2% (383/779)<br><br>Of those:<br>- UC: 91.9% (349/383)<br>- OC: 8.9% (34/383)	59.4% (528/889)<br><br>Of those:<br>- A: 38.6%* (204/528)<br>- S: 51.9% (274/528)<br>- I: 9.47% (50/528)	Oral formulations more often within the anticipated concentration than parental formulations<br><br>Defined range of declared label: 50-200%<br><br>Label information:<br>204 different manufacturers in 48 different countries origin<br><br>Possible over- or under estimation of qualitative analysis due to mixed sample (adulterated and original)
Fabresse N., et.al., 2021, France [2]	2016-2019	GC-MS,<br>LC-HRMS,<br>LC-MS/MS	Seized pharmaceuticals by Justice in sport hall	S1, S2, S4, sexual performance enhancers, Dietary supplements<br>Total N=75<br><br>S1 N= 54 (72%)	7.41%** (4/54)<br><br>20.4%* (11/54)	37.0% (20/54)	40.7% (22/54)	8/54 not quantified, of those 7/54 qualitatively accurately labeled<br><br>Defined range of declared label 80-130%<br><br>Other substance classes not further specified
Neves D., et. al. 2013, Brazil [3]	2006-2011	GC-MS,<br>IR/UV spectrophotometry,<br>LC-HRMS	Seized pharmaceuticals by the Brazilian Federal Police department	S1, S2, S3,<br><br>Total N=3676<br><br>S1: N=3636 (98.9%)<br><br>N=2818/3676 (76.7%) chemically analyzed	58.6%* (1651/2818)		41.4% (1167/2818)<br><br>Of those:<br>- A: 7.03%* (82/1167)<br>- S: 28.3% (330/1167)<br>- I: 48.6%* (567/1167)<br>- Fake packaging/ manufacturer 16.1% (188/1167)	Mixed sample<br><br>Other substance classes not further specified<br><br>Increase by 5.2-fold (1468/ 282) of seizures from 2007-2011<br><br>No analysis of concentrations was conducted

Odoardi A., et. al., 2021, Italy [4]	2017-2019	GC-MS,<br>LC-HRMS	Confiscated by police operations performed by Carabinieri in the whole Italian territory	S1, S3, S4, sexual performance enhancers, Stimulants<br><br>Total N=409<br>S1: N=267 (65%)<br><br>S4: N=49 (12%)	74.2%* (198/267)		19.5% (52/267)	Not labelled S1: 6.37% (17/267)<br><br>Quantitative (N=unknown) determination showed discrepancies between declared and actual content. For AAS, the quantitative content of the single compound was always lower than the one stated on the label
Krug O., et. al., 2014, Germany [5]	2010-2013	LC-HRMS,<br>GC-HRMS,<br>SDS-PAGE coupled with nano LC-HRMS	Confiscated products by the German Bureau of Customs Investigation or purchased on the black market via internet	S1, S2, S4, Others<br><br>Total N=337<br>S1: 83.7%<br>S2: 12.8%<br>S4: 3.2%	43%*		57%	Mixed sample<br><br>No analysis of concentrations was conducted
Neves D., et. al. 2017, Brazil [6]	2011-2016	GC-MS	Seized pharmaceuticals by the Brazilian Federal Police department	S1, Dietary supplements<br><br>S1: N=328	47.0%** (154/328)<br><br>57.9%* (190/328)	11.0% (36/328)<br><br>Of those:<br>- OC: 58.3% (21/36)<br>- UC: 41.7% (15/36)	42.1% (138/328)*<br><br>Of those:<br>- A: 1.45% (2/138)<br>- S: 29.7% (41/138)<br>- I: 52.2% (72/138)<br>- Inexistent medication 9.42% (13/138)	Counterfeit: Differences in formulation: 28.7% tablets, 12% suspension, 65% oil-based solutions<br><br>OC (142% up to 221% of label; most likely fake in oil-based solutions).<br>UC (16.4%-58% of label; most likely fake in oil-based solutions)
Graham M., et. al., 2009, United Kingdom, [7]	2009	GC-MS	Black market	S1, S2, S3, S4, S5<br><br>N= 57 (78.9% S1)<br><br>S1: N= 42	59.5%* (25/42)		42.5% (17/42)<br><br>Of those:<br>S: 41.2% (7/17)<br>I: 58.8% (10/17)<br>A: 0% (0/17)	No analysis of concentrations was conducted<br><br>Skin commensals were found as contaminants in injectables (quantity of vials unknown)<br><br>More counterfeiting in injectables vs. oral formulations
Tircova B., et. al., 2019, Czech Republic/ Slovakia, [8]	2017-2018	LC-MS/MS	Obtained voluntarily from users	S1<br><br>N=358	59.2%** (212/358)<br><br>79.6%* (285/358)	20.4% (73/358)<br><br>Of those:<br>- OC: 17.8% (13/73)	20.4% (73/358)<br><br>Of those:<br>- S: 21.9% (16/73)<br>- I: 78.1% (57/73)	Defined range of declared label 80-120%<br><br>Differences in formulation:

						- UC: 82.2% (60/73)	- A: n.d.	43% of oil-based substances vs. 37% of tablets and capsules were substandard or counterfeit<br><br>Label information:<br>48 different manufacturers, 29 underground labs, 19 pharmaceutical companies
Thevis M. et. al., 2008, Germany, [9]	Un-known	LC-MS/MS, GC-MS	Confiscated products	S1, S4, Stimulants, Sexual performance enhancements<br><br>N=70<br>S1: N= 50 (68.6%)	68.0%* (34/50)		32.0% (16/50)<br><br>S: 62.5% (10/16)<br>A: 37.5% (6/16)<br>I: 0% (0/16)	Visual inspection did not allow a differentiation between original and counterfeit products<br><br>No analysis of concentrations was conducted
Ribeiro M., et. al., 2018, Brazil, [10]	Un-known	1H NMR, GC-MS	Seized pharmaceuticals by the Brazilian Federal Police department	S1<br><br>N=40	22.5%** (9/40)<br><br>80%* (32/40)	57.5% (23/40)	20%* (8/40)	Quantitative results (without defined range): substandard mostly under-concentrated
Berneira L., et. al., 2019, Brazil, [11]	2017	GC-MS, FT-IR, DSC	Seized pharmaceuticals by the Brazilian Federal Police department	S1<br><br>N=8	75%* (6/8)		25% (2/8)	Visual inspection allied with instrumental characterization of the AAS, were crucial procedures to characterize and detect falsifications<br><br>Quantitative results (without defined range): substandard all under-concentrated
Coopman V., et. al., 2012, Belgium, [12]	Un-known	LC-UV, GC-MS	Confiscated products from bodybuilders	S1, S3, S4, sexual performance enhancements<br>N=71<br><br>S1: N=56 (56.3%)	60.7%* (34/56)*		39.3% (22/56)<br><br>Of those:<br>- S: 54.5% (12/22)<br>- A: 40.9% (9/22)<br>- I: 4.55% (1/22)	*No analysis of concentrations was conducted, but quantities are described to be lower
Pellegrini M., et. al., 2012, Italy, [13]	Un-known	GC-MS	Seized by Carabinieri	S1<br><br>N=15	13.3%** (2/15)<br><br>33.3%* (5/15)	20% (3/15)	66.7% (10/15)<br><br>Of those:<br>- S: 80% (8/10)<br>- I: 20% (2/10)<br>- A: 0% (0/10)	No range of concentrations declared for substandard declared by author

Riberio M., et. al., 2018, Brazil, [14]	Un-known	1H NMR spectroscopic	Commercial samples	S1<br><br>N=16	75%* (12/16)		25% (4/16)	For the majority of analyzed drugs the concentration was much smaller than indicated
Campos E., et. al., 2020, Brazil, [15]	Un-known	LC-HRMS	Seized pharmaceuticals by the Brazilian Federal Police department	S1<br>Only tablet/packs of Stanozolol<br><br>N=31	9.68%** (3/31)	90.3% (28/31)<br><br>Of those:<br>- OC: 71.4% (20/28)<br>- UC: 28.6% (8/28)		Stanozolol was tested for quantity only; Defined range of declared label was between 90-110% (Lowest concentration found 9.3% (23.3/250mg) of indicated on label, highest concentration found 225% (22.5mg/10mg).
Musshoff, F. et. al. 1997, Germany [16]	Un-known	GC-MS	Black market	S1<br><br>N=37 known AAS	59.5%* (22/37)		40.5% (15/37)<br><br>Of those:<br>- S: 93.3% (14/15)<br>- A: 0% (0/15)<br>- I: 6.67% (1/15)	No analysis of concentrations was conducted
Lemos, V. F. et. al. 2021, Brazil [17]	Un-known	Attenuated Total Reflection Fourier Transform Infrared Microspectroscopy (μATR-FTIR) mapping	Seized pharmaceuticals by the Brazilian Federal Police department	S1<br><br>N=30	70%* (21/30)		30% (9/30)<br><br>Of those:<br>- S: 33.3% (3/9)<br>- A: 22.2% (2/9)<br>- I: 44.4% (4/9)	Tablets only<br><br>No analysis of concentrations was conducted
Forsdahl, G. et. al. 2011, Austria [18]	Un-known	GC-MS	Seized pharmaceuticals by the Austrian police	S1<br><br>N=28 known testosterone esters	71.4%* (20/28)		28.6% (8/28)<br><br>Of those:<br>- S: 0% (0/8)<br>- A: 87.5% (7/8)<br>- I: 12.5% (1/8)	Oil-based testosterone esters for injection only<br><br>No analysis of concentrations was conducted
Kozlik, P. et. al. 2016, Slovakia [19]	Un-known	LC-MS/MS	Blackmarket (online, dealer)	S1<br><br>N=9	88.9%* (8/9)<br><br>66.7%** (6/9)	22.2% (2/9)	11.1% (1/9)	Oil-based testosterone esters for injection only

*Only accurately labelled/qualitative analysis only
**Accurately labelled and concentration within range/qualitative and quantitative analysis
S = Substituted
A= Adulterated
I = Inert
OC = Overconcentrated
UC = Underconcentrated
n.d = not declared

Bibliography

1. Weber C, Krug O, Kamber M, Thevis M: Qualitative and Semiquantitative Analysis of Doping Products Seized at the Swiss Border. *Subst Use Misuse* 2017, 52(6):742-753.
2. Fabresse N, Gheddar L, Kintz P, Knapp A, Larabi IA, Alvarez JC: Analysis of pharmaceutical products and dietary supplements seized from the black market among bodybuilders. *Forensic Sci Int* 2021, 322:110771.
3. da Justa Neves DB, Marcheti RG, Caldas ED: Incidence of anabolic steroid counterfeiting in Brazil. *Forensic Sci Int* 2013, 228(1-3):e81-83.
4. Odoardi S, Mestria S, Biosa G, Valentini V, Federici S, Strano Rossi S: An overview on performance and image enhancing drugs (PIEDs) confiscated in Italy in the period 2017-2019. *Clin Toxicol (Phila)* 2021, 59(1):47-52.
5. Krug O, Thomas A, Walpurgis K, Piper T, Sigmund G, Schänzer W, Laussmann T, Thevis M: Identification of black market products and potential doping agents in Germany 2010-2013. *Eur J Clin Pharmacol* 2014, 70(11):1303-1311.
6. Neves DBDJ, Caldas ED: GC-MS quantitative analysis of black market pharmaceutical products containing anabolic androgenic steroids seized by the Brazilian Federal Police. *Forensic Sci Int* 2017, 275:272-281.
7. Graham MR, Ryan P, Baker JS, Davies B, Thomas NE, Cooper SM, Evans P, Easmon S, Walker CJ, Cowan D *et al*: Counterfeiting in performance- and image-enhancing drugs. *Drug Test Anal* 2009, 1(3):135-142.
8. Tircova B, Bosakova Z, Kozlik P: Development of an ultra-high performance liquid chromatography-tandem mass spectrometry method for the determination of anabolic steroids currently available on the black market in the Czech Republic and Slovakia. *Drug Test Anal* 2019, 11(2):355-360.
9. Thevis M, Schrader Y, Thomas A, Sigmund G, Geyer H, Schänzer W: Analysis of confiscated black market drugs using chromatographic and mass spectrometric approaches. *J Anal Toxicol* 2008, 32(3):232-240.
10. Ribeiro MVM, Boralle N, Felippe LG, Pezza HR, Pezza L: H NMR determination of adulteration of anabolic steroids in seized drugs. *Steroids* 2018, 138:47-56.
11. Berneira LM, de Freitas SC, da Silva CC, Machado AM, de Pereira CMP, Dos Santos MAZ: Application of differential scanning calorimetry in the analysis of apprehended formulations of anabolic androgenic steroids. *Forensic Sci Int* 2019, 296:15-21.
12. Counterfeit drugs and pharmaceutical preparations seized from the black market among bodybuilders, Vera Coopman, Jan Cordonnier, Ann Toxicol Anal 24 (2) 73-80 (2012), DOI: 10.1051/ata/2012012. In.
13. A simple toxicological analysis of anabolic steroid preparations from the black market, Manuela Pellegrini, Maria Concetta Rotolo, Rita Di Giovannadrea, Roberta Pacifici, Simona Pichini, Ann Toxicol Anal 24 (2) 67-72 (2012), DOI: 10.1051/ata/2012011. In.
14. Ribeiro MV, Boralle N, Pezza HR, Pezza L. Authenticity assessment of anabolic androgenic steroids in counterfeit drugs by 1 H NMR. Analytical Methods. 2018;10(10):1140-50. In.

15. Campos ER, Eller S, Birk L, Coimbra MA, Macedo SM, Yonamine M, Merib JO, de Oliveira TF. Analysis of seized stanozolol formulations in South Brazil by liquid chromatography coupled to quadrupole time-of-flight-mass spectrometry. Drug Analytical Research. 2020 Dec 18;4(2):58-63. In.
16. Musshoff F, Daldrup T, Ritsch M: [Anabolic steroids on the German black market]. *Arch Kriminol* 1997, 199(5-6):152-158.
17. Lemos VF, Ortiz RS, Limberger RP: Forensic analysis of anabolic steroids tablets composition using attenuated total reflection Fourier transform infrared microspectroscopy (μATR-FTIR) mapping. *J Forensic Sci* 2021, 66(3):837-845.
18. Forsdahl G, Östreicher C, Koller M, Gmeiner G: Carbon isotope ratio determination and investigation of seized testosterone preparations. *Drug Test Anal* 2011, 3(11-12):814-819.
19. Kozlik P, Tircova B: Development of the fast, simple and fully validated high performance liquid chromatographic method with diode array detector for quantification of testosterone esters in an oil-based injectable dosage form. *Steroids* 2016, 115:34-39.
