# Supplementary material for: Fake anabolic androgenic steroids on the black market – a systematic review and meta-analysis on qualitative and quantitative analytical results found within the literature
Source: BMC Public Health. 2022 Jul 17;22:1371. doi: 10.1186/s12889-022-13734-4 (PMC9288681; doi:10.1186/s12889-022-13734-4)
Supplement: Supplementary file 4 — Additional file 4. Main outcome analyses. [file 12889_2022_13734_MOESM4_ESM.pdf]

# Fake Anabolic Androgenic Steroids on the Black Market – a systematic review and meta-analysis

## MAIN OUTCOME 1:
## Proportions of counterfeit AAS-samples

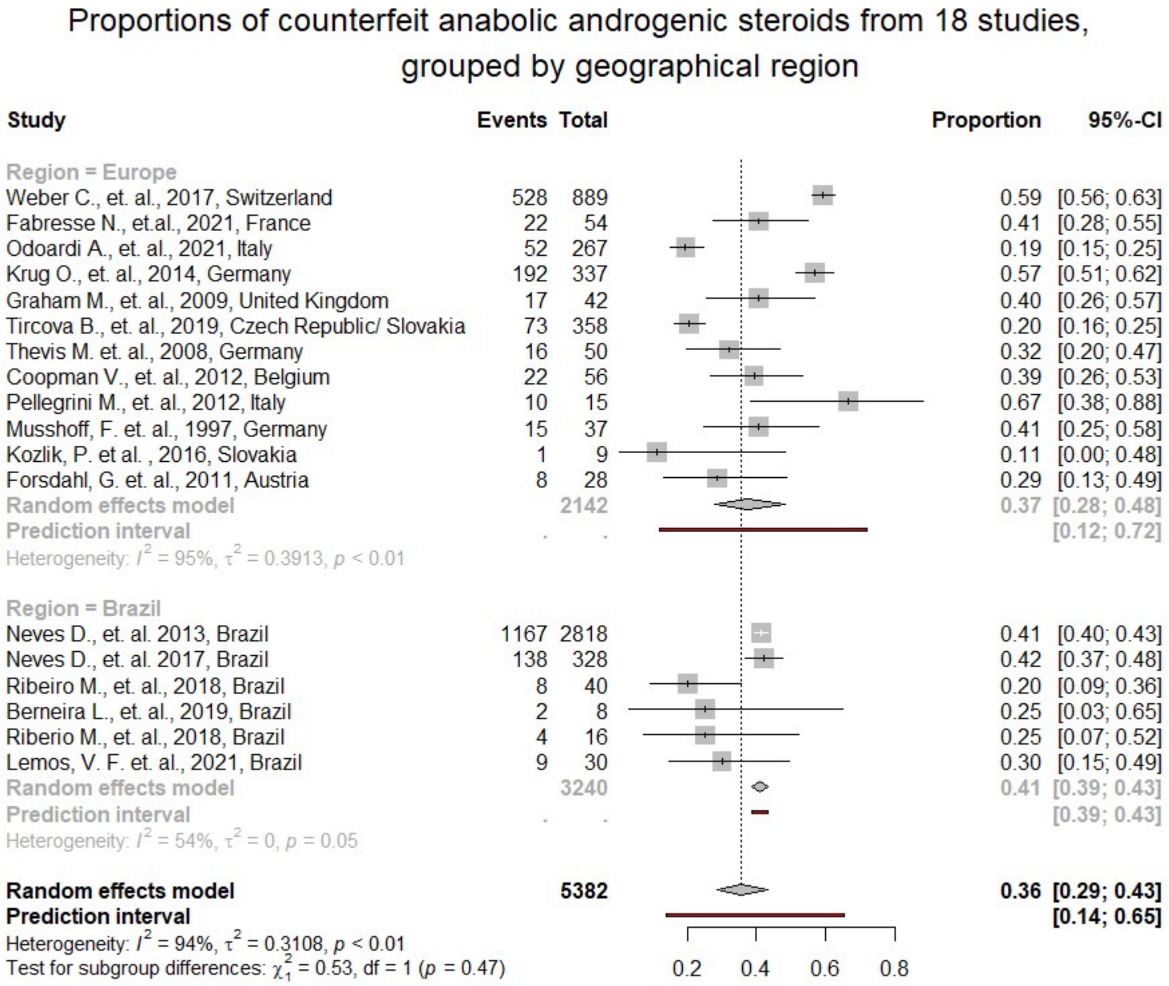


Number of studies combined: k = 18
Number of observations: o = 5382
Number of events: e = 2284

| | proportion | 95%-CI |
|---|---|---|
| Random effects model | 0.3573 | [0.2869; 0.4346] |
| Prediction interval | | [0.1403; 0.6545] |

Quantifying heterogeneity:

tau^2 = 0.3108; tau = 0.5575; I^2 = 93.7% [91.4%; 95.4%]; H = 3.98 [3.41; 4.65]

## Test of heterogeneity:

Q d.f. p-value Test
269.34 17 < 0.0001 Wald-type
301.17 17 < 0.0001 Likelihood-Ratio

## Results for subgroups (random effects model):

k proportion 95%-CI tau^2 tau Q I^2
Region = Europe 12 0.3742 [0.2770; 0.4828] 0.3913 0.6256 244.19 95.5%
Region = Brazil 6 0.4099 [0.3879; 0.4323] 0 0 10.94 54.3%

## Test for subgroup differences (random effects model):

Q d.f. p-value
Between groups 0.53 1 0.4662

## Prediction intervals for subgroups:

95%-PI
Region = Europe [0.1214; 0.7213]
Region = Brazil [0.3861; 0.4341]

Details on meta-analytical method:
- Random intercept logistic regression model
- Maximum-likelihood estimator for tau^2
- Hartung-Knapp adjustment for random effects model
- Logit transformation
- Clopper-Pearson confidence interval for individual studies

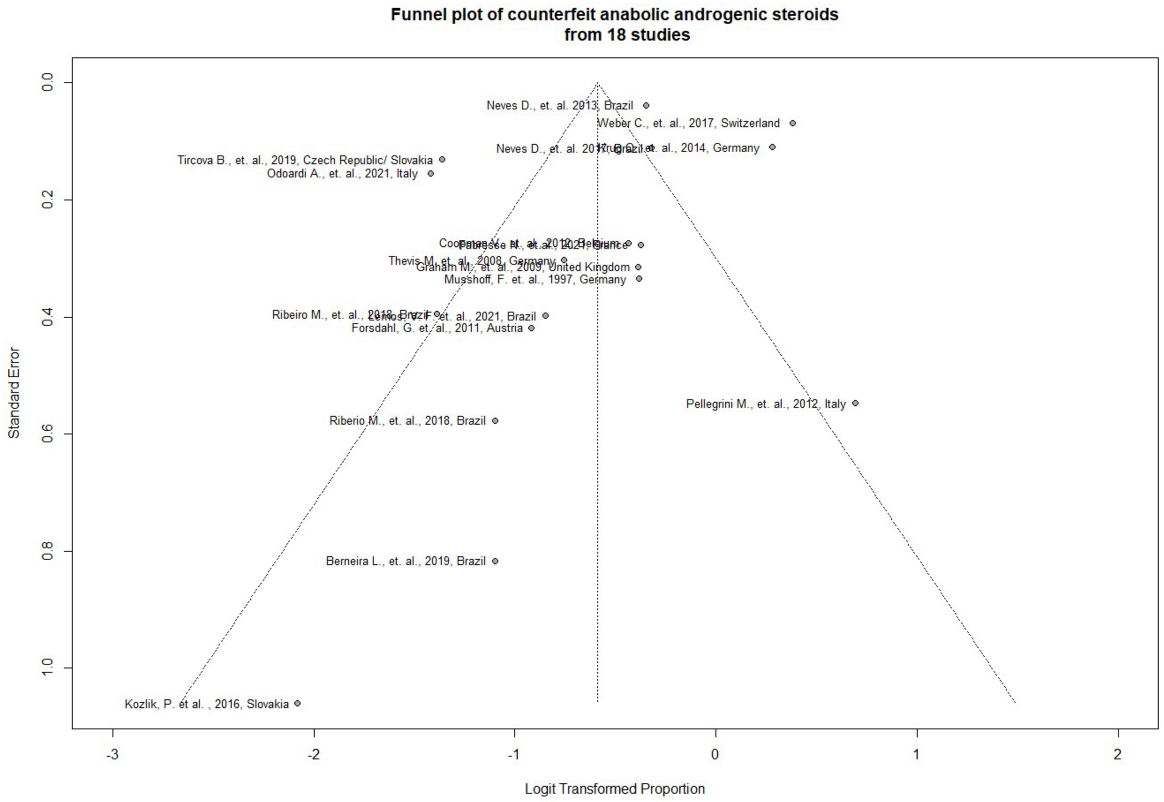


## Review: Counterfeit

## Linear regression test of funnel plot asymmetry

Test result: t = -0.80, df = 16, p-value = 0.4365

Sample estimates:
    bias se.bias intercept se.intercept
 -10.3550 12.9735   -0.2476       0.1206

Details:
- multiplicative residual heterogeneity variance (tau^2 = 16.1889)
- predictor: inverse of total sample size
- weight:    inverse variance of average event probability
- reference: Peters et al. (2006), JAMA

## Meta-regression by publication year

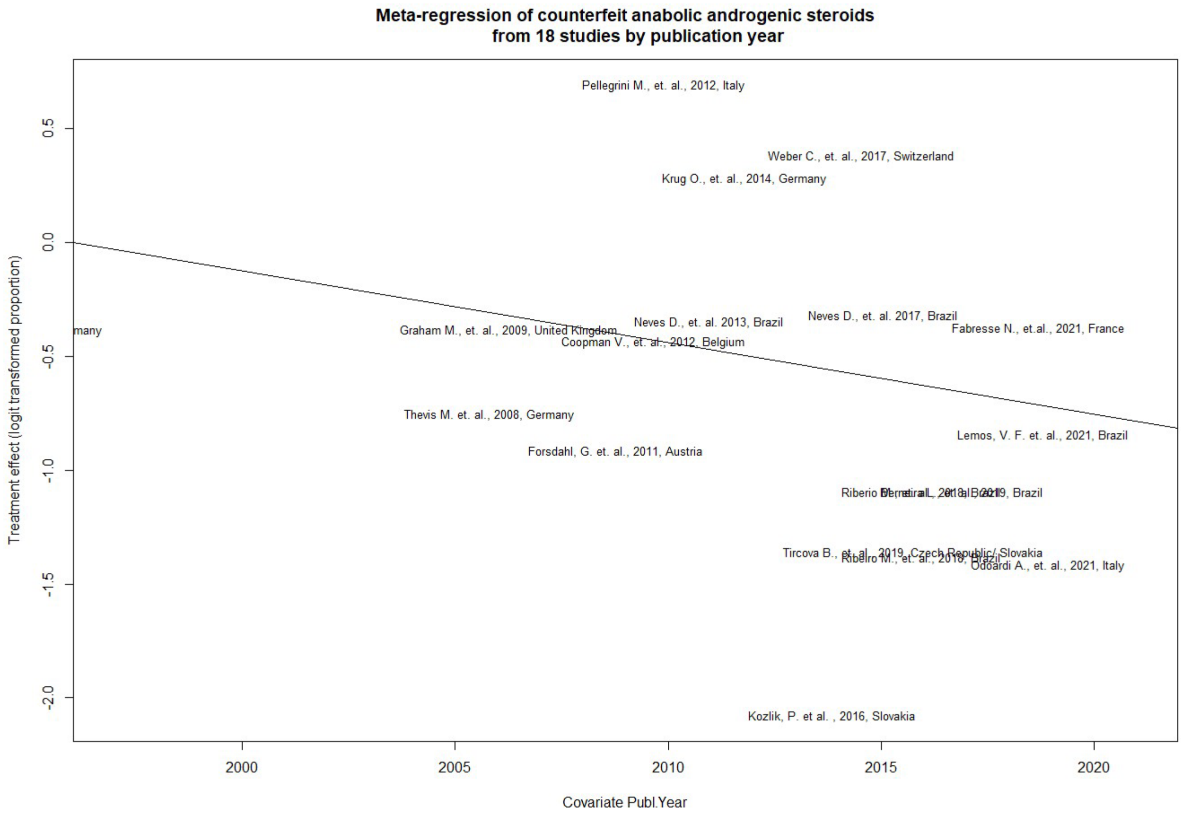


Mixed-Effects Model (k = 18; tau^2 estimator: ML)

tau^2 (estimated amount of residual heterogeneity):     0.2842
tau (square root of estimated tau^2 value):             0.5331
I^2 (residual heterogeneity / unaccounted variability): 92.2615%
H^2 (unaccounted variability / sampling variability):   12.9224

Tests for Residual Heterogeneity:
Wld(df = 16) = 265.3596, p-val < .0001
LRT(df = 16) = 289.6485, p-val < .0001

Test of Moderators (coefficient 2):
F(df1 = 1, df2 = 16) = 1.5458, p-val = 0.2317

Model Results:

| | estimate | se | tval | df | pval | ci.lb | ci.ub |
|---|---|---|---|---|---|---|---|
| intrcpt | 62.8358 | 51.0089 | 1.2319 | 16 | 0.2358 | -45.2983 | 170.9699 |
| Publ.Year | -0.0315 | 0.0253 | -1.2433 | 16 | 0.2317 | -0.0852 | 0.0222 |

---
Signif. codes: 0 ‘***’ 0.001 ‘**’ 0.01 ‘*’ 0.05 ‘.’ 0.1 ‘ ’ 1

# MAIN OUTCOME 2:
# Proportions of substandard AAS-samples

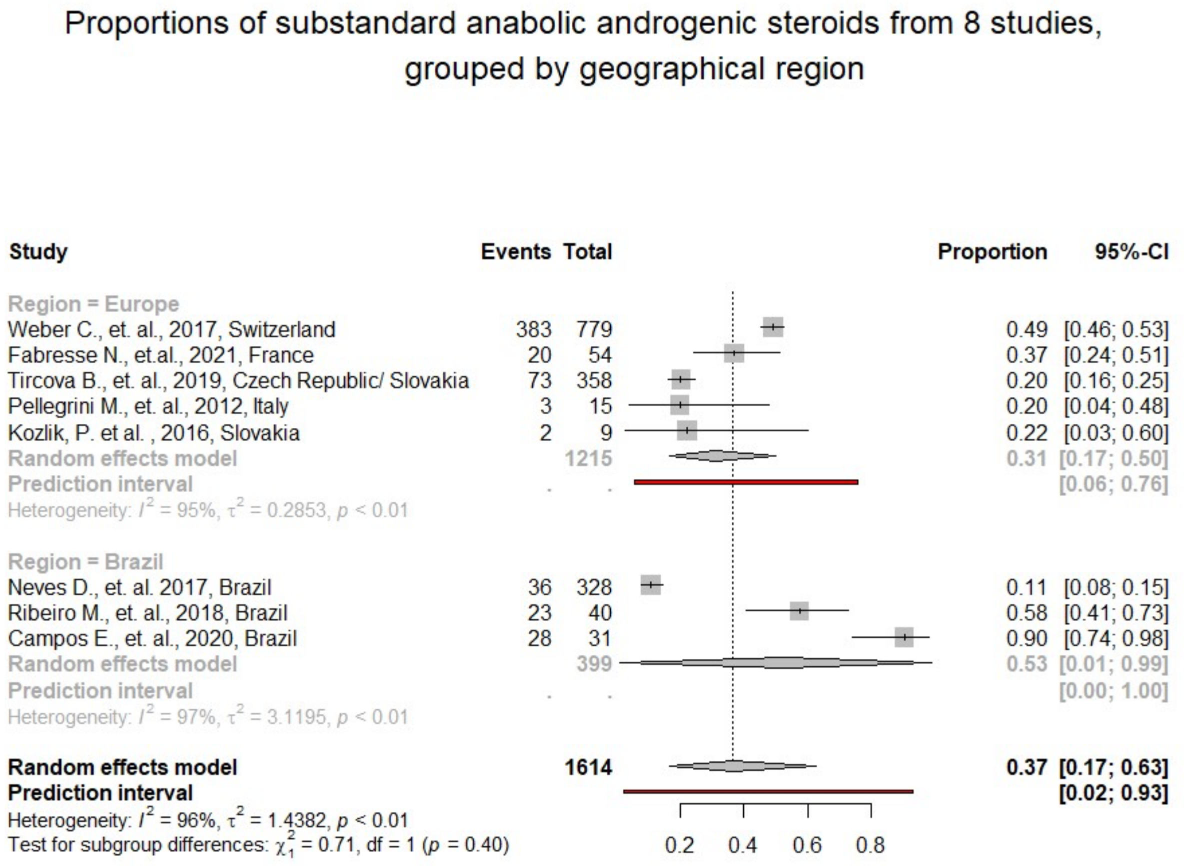


Number of studies combined: k = 8

Number of observations: o = 1614

Number of events: e = 568

| | proportion | 95%-CI |
|---|---|---|
| Random effects model | 0.3683 | [0.1681; 0.6273] |
| Prediction interval | | [0.0248; 0.9304] |

### Quantifying heterogeneity:

tau^2 = 1.4382; tau = 1.1993; I^2 = 96.4% [94.7%; 97.6%]; H = 5.29 [4.33; 6.48]

### Test of heterogeneity:

| Q | d.f. | p-value | Test |
|---|---|---|---|
| 196.24 | 7 | < 0.0001 | Wald-type |
| 254.95 | 7 | < 0.0001 | Likelihood-Ratio |

### Results for subgroups (random effects model):

| | k | proportion | 95%-CI | tau^2 | tau | Q | I^2 |
|---|---|---|---|---|---|---|---|
| Region = Europe | 5 | 0.3096 | [0.1664; 0.5017] | 0.2853 | 0.5341 | 83.25 | 95.2% |

Region = Brazil 3 0.5289 [0.0123; 0.9902] 3.1195 1.7662 79.09 97.5%

Test for subgroup differences (random effects model):

Q d.f. p-value

**Between groups 0.71 1 0.3981**

Prediction intervals for subgroups:

**95%-PI**

Region = Europe [0.0607; 0.7566]

Region = Brazil [0.0000; 1.0000]

Details on meta-analytical method:

- Random intercept logistic regression model
- Maximum-likelihood estimator for tau^2
- Hartung-Knapp adjustment for random effects model
- Logit transformation
- Clopper-Pearson confidence interval for individual studies

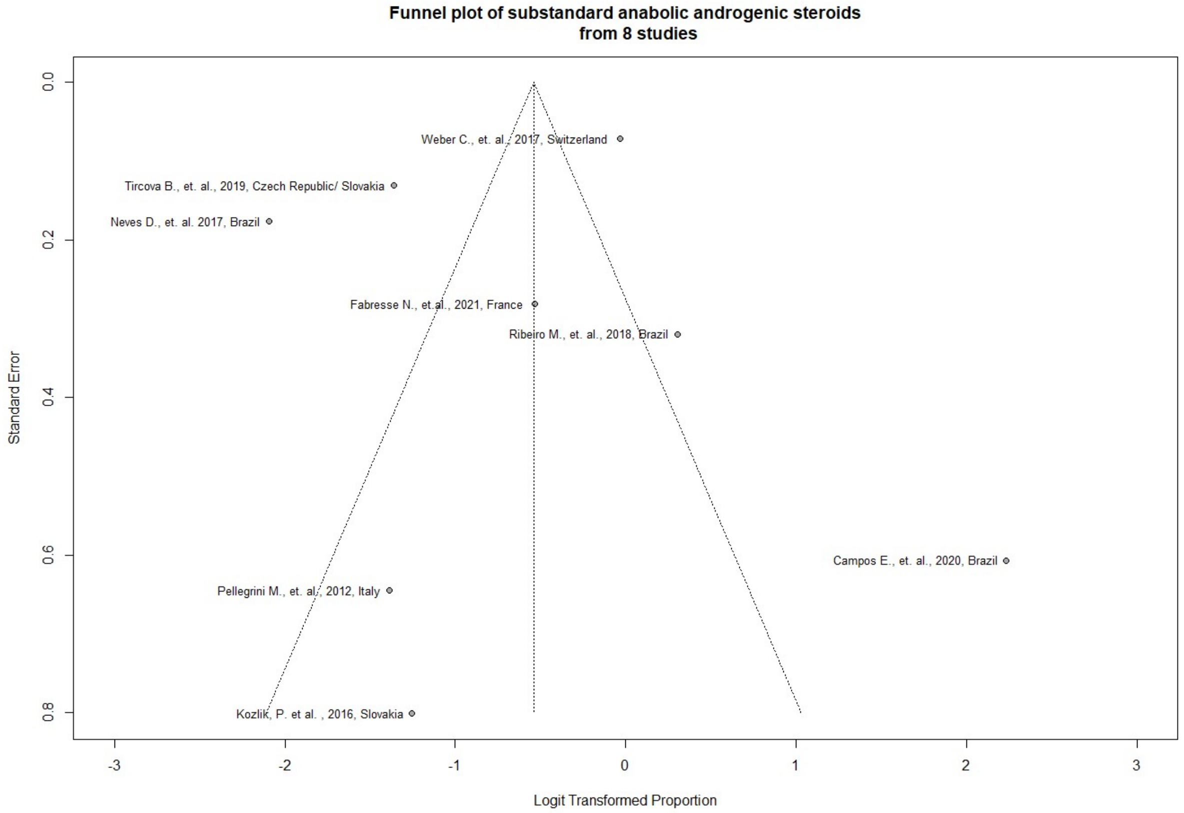


Review: Substandard

Linear regression test of funnel plot asymmetry

Test result: t = -0.03, df = 6, p-value = 0.9803

Sample estimates:

| bias | se.bias | intercept | se.intercept |
|---|---|---|---|
| -0.7573 | 29.3840 | -0.4926 | 0.3486 |

Details:
- multiplicative residual heterogeneity variance (tau^2 = 32.7035)
- predictor: inverse of total sample size
- weight: inverse variance of average event probability
- reference: Peters et al. (2006), JAMA

## Meta-regression by publication year

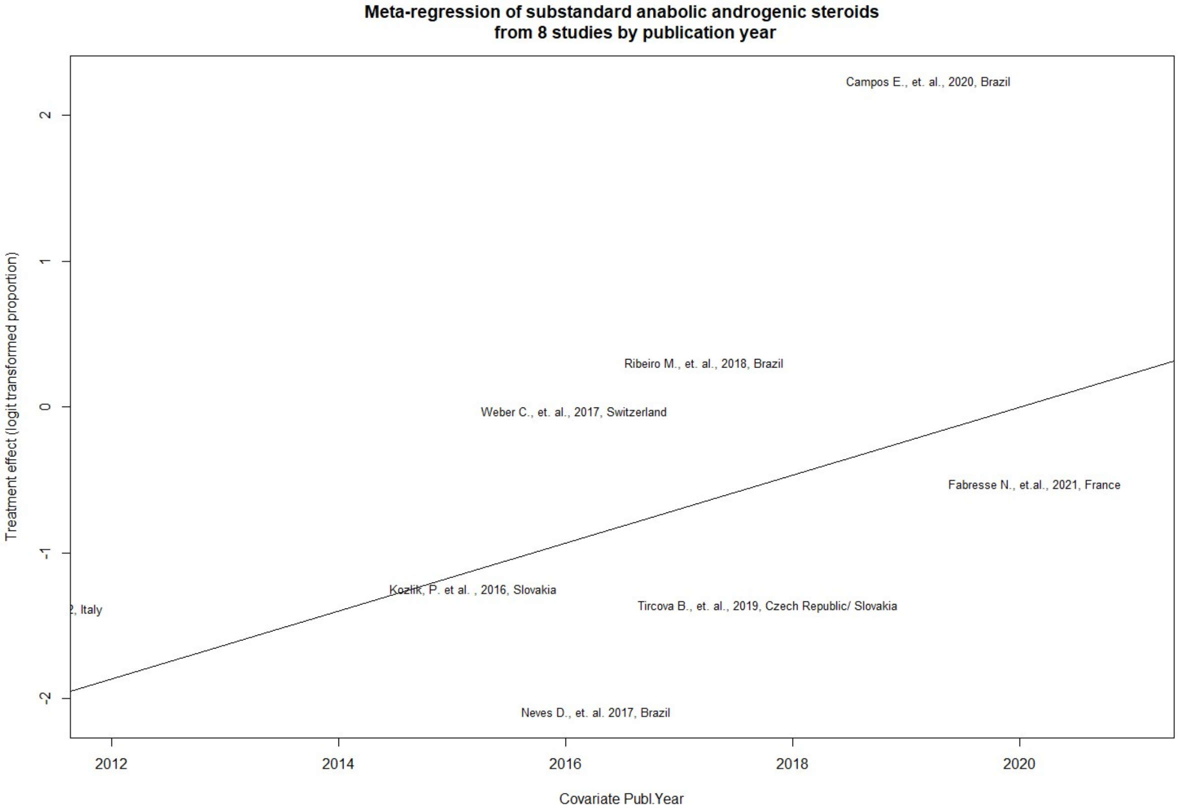


## Mixed-Effects Model (k = 8; tau^2 estimator: ML)

tau^2 (estimated amount of residual heterogeneity): 1.1616
tau (square root of estimated tau^2 value): 1.0778
I^2 (residual heterogeneity / unaccounted variability): 96.1925%
H^2 (unaccounted variability / sampling variability): 26.2642

## Tests for Residual Heterogeneity:

Wld(df = 6) = 187.8444, p-val < .0001
LRT(df = 6) = 253.3894, p-val < .0001

## Test of Moderators (coefficient 2):

F(df1 = 1, df2 = 6) = 1.9481, p-val = 0.2123

## Model Results:

estimate se tval df pval ci.lb ci.ub

| | | | | | | | |
|---|---|---|---|---|---|---|---|
| intrcpt | -469.6791 | 336.1271 | -1.3973 | 6 | 0.2118 | -1292.1525 | 352.7943 |
| Publ.Year | 0.2325 | 0.1666 | 1.3957 | 6 | 0.2123 | -0.1751 | 0.6401 |

---

Signif. codes: 0 ‘***’ 0.001 ‘**’ 0.01 ‘*’ 0.05 ‘.’ 0.1 ‘ ’ 1
