# Supplementary material for: Fake anabolic androgenic steroids on the black market – a systematic review and meta-analysis on qualitative and quantitative analytical results found within the literature
Source: BMC Public Health. 2022 Jul 17;22:1371. doi: 10.1186/s12889-022-13734-4 (PMC9288681; doi:10.1186/s12889-022-13734-4)
Supplement: Supplementary file 5 — Additional file 5. Supplementary analyses. [file 12889_2022_13734_MOESM5_ESM.pdf]

Fake Anabolic Androgenic Steroids on the Black Market – a systematic review and meta-analysis

SUPPLEMENTAL MATERIAL

S 1.1. PROPORTION OF INERT AAS-SAMPLES

SUMMARY
INERT AAS-SAMPLES (No active ingredients):
24% (CI-95: 0.09 T0 0.49) OVERALL; WITH HIGH HETEROGENEITY (96%); SIGNIFICANTLY LOWER (p < 0.05) IN EUROPE (15%) THAN IN BRAZIL (49%).
Meta-Regression by publication year showed a significant increase of inert AAS-samples over time (p < 0.05).

Review: **Inert**

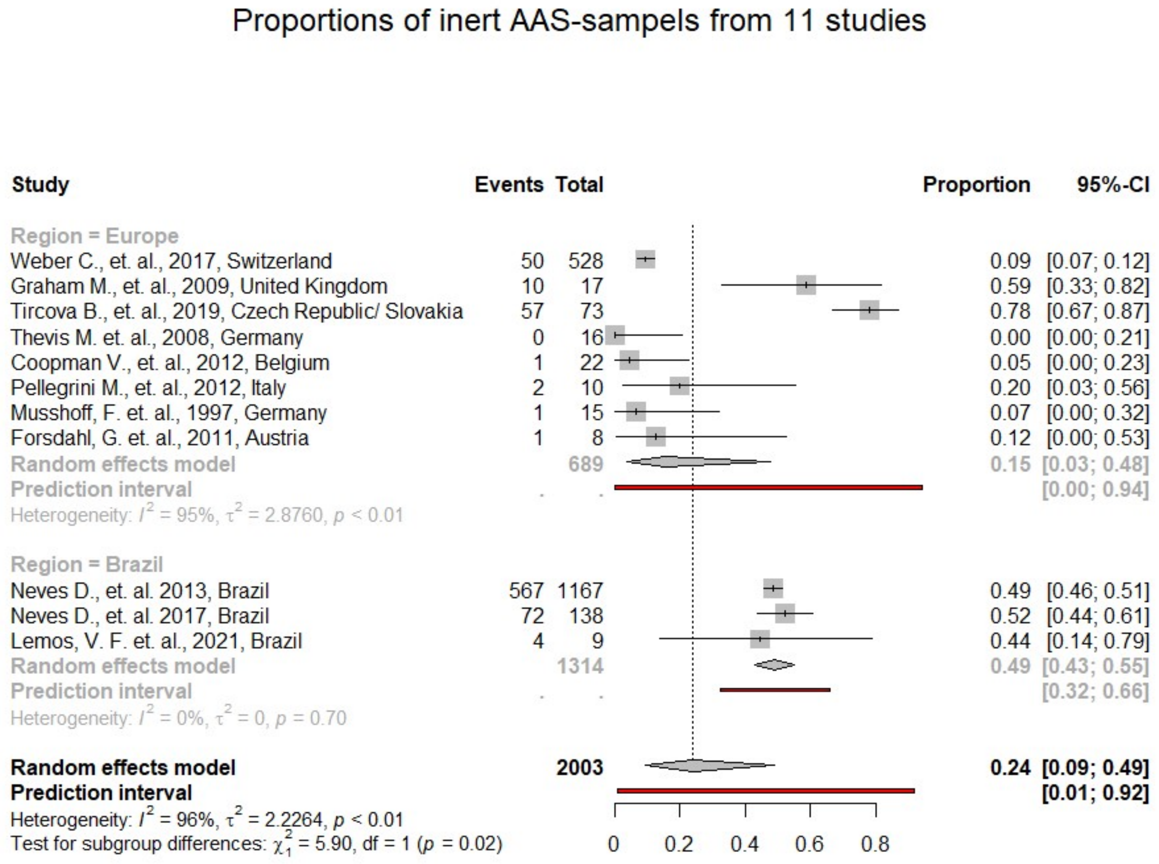

Number of studies combined: k = 11
Number of observations: o = 2003
Number of events: e = 765

	proportion	95%-CI
Random effects model	0.2385	[0.0927; 0.4899]
Prediction interval		[0.0088; 0.9169]

Quantifying heterogeneity:

tau^2 = 2.2264; tau = 1.4921**; I^2 = 95.9% [94.2%; 97.1%]**; H = 4.95 [4.15; 5.90]

Test of heterogeneity:

Q	d.f.	p-value	Test
244.79	10	< 0.0001	Wald-type
381.56	10	< 0.0001	Likelihood-Ratio

Results for subgroups (random effects model):

	k	proportion	95%-CI	tau^2	tau	Q	I^2
Region = Europe	8	0.1535	[0.0348; 0.4769]	2.8760	1.6959	139.34	95.0%
Region = Brazil	3	0.4893	[0.4304; 0.5486]	0	0	0.71	0.0%

Test for subgroup differences (random effects model):

	Q	d.f.	p-value
**Between groups**	**5.90**	**1**	**0.0151**

Prediction intervals for subgroups:

	95%-PI
Region = Europe	[0.0021; 0.9408]
Region = Brazil	[0.3222; 0.6589]

Details on meta-analytical method:

- Random intercept logistic regression model
- Maximum-likelihood estimator for tau^2
- Hartung-Knapp adjustment for random effects model
- Logit transformation
- Clopper-Pearson confidence interval for individual studies
- Continuity correction of 0.5 in studies with zero cell frequencies
  (only used to calculate individual study results)

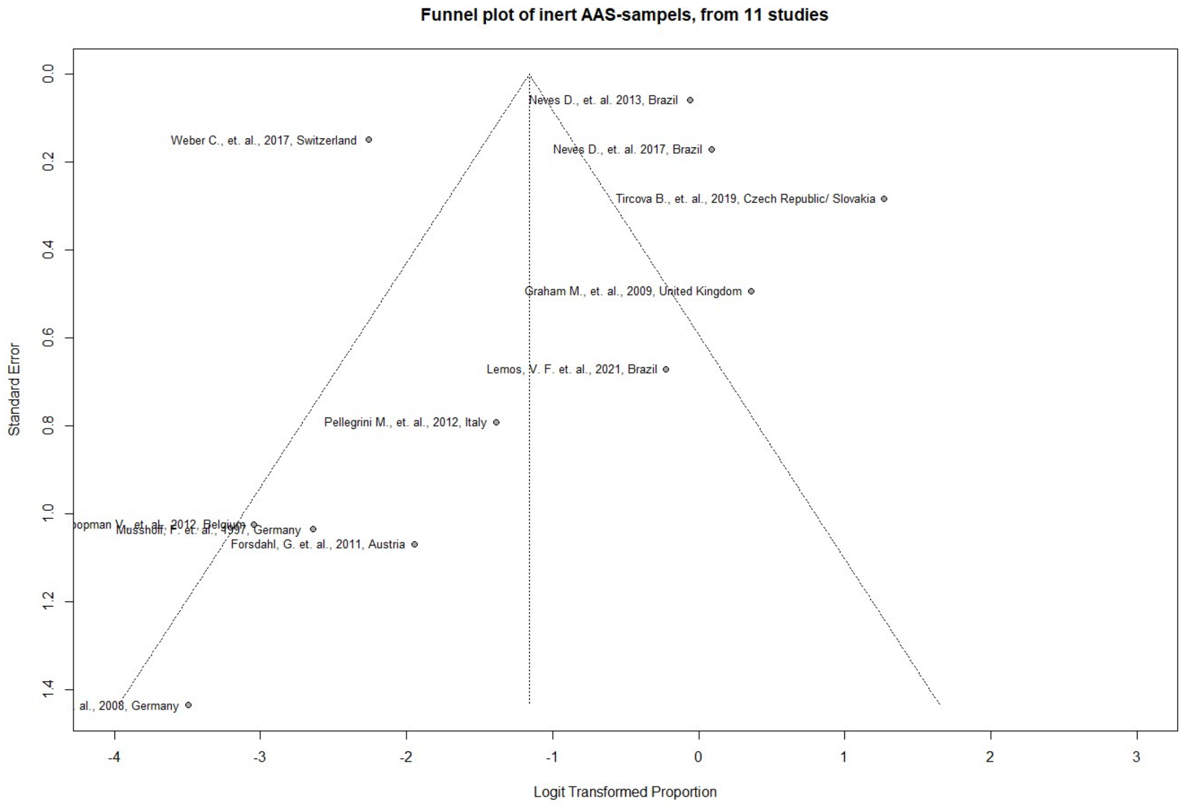

Linear regression test of funnel plot asymmetry

Test result: t = -0.15, df = 9, p-value = 0.8858

Sample estimates:

bias	se.bias	intercept	se.intercept
-2.9725	20.1245	-0.2619	0.2902

Details:
- multiplicative residual heterogeneity variance (tau^2 = 30.5153)
- predictor: inverse of total sample size
- weight: inverse variance of average event probability
- reference: Peters et al. (2006), JAMA

Meta-regression by publication year

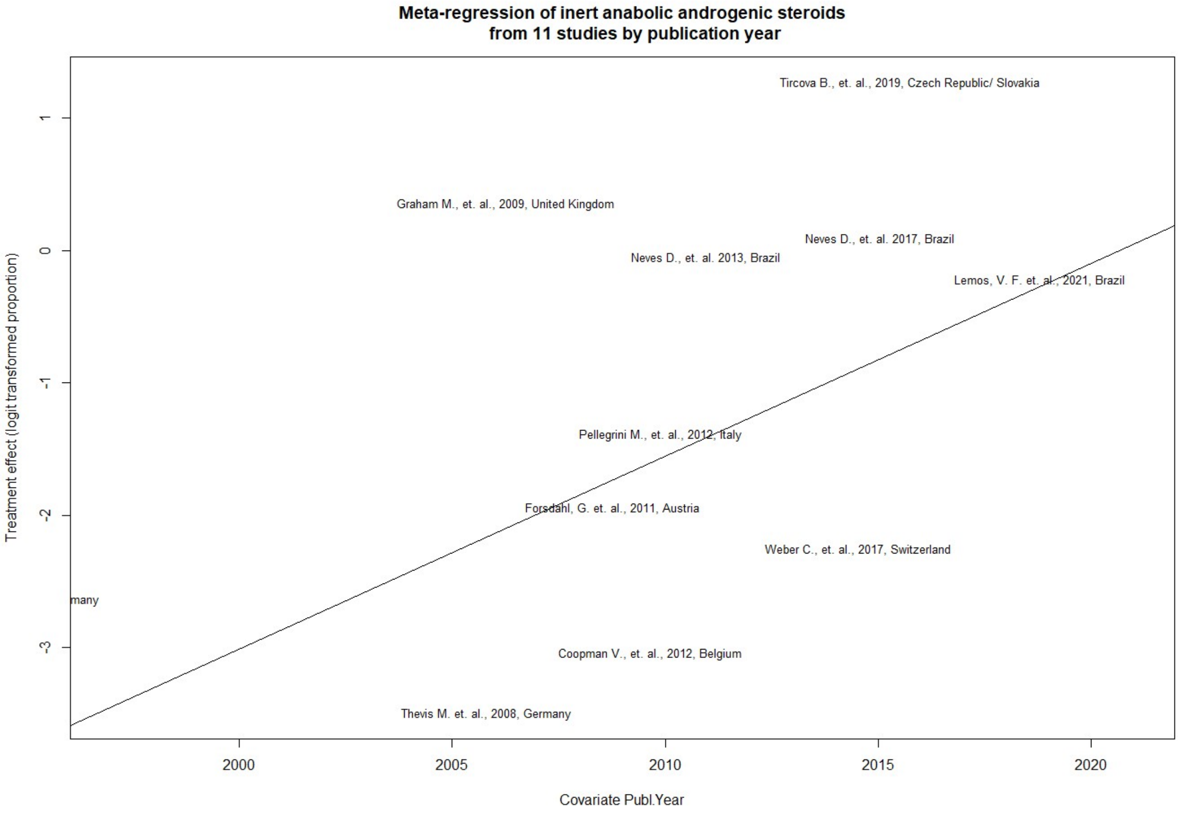

Mixed-Effects Model (k = 11; tau^2 estimator: ML)

tau^2 (estimated amount of residual heterogeneity): 1.5954
tau (square root of estimated tau^2 value): 1.2631
I^2 (residual heterogeneity / unaccounted variability): 95.1459%
H^2 (unaccounted variability / sampling variability): 20.6013

Tests for Residual Heterogeneity:
Wld(df = 9) = 230.6491, p-val < .0001
LRT(df = 9) = 364.3765, p-val < .0001

Test of Moderators (coefficient 2):
F(df1 = 1, df2 = 9) = 3.6433, p-val = 0.0886

Model Results:

	estimate	se	tval	df	pval	ci.lb	ci.ub	
intrcpt	-294.2141	153.5846	-1.9156	9	0.0877	-641.6467	53.2185	.
Publ.Year	0.1456	0.0763	1.9088	9	0.0886	-0.0270	0.3182	.

Signif. codes: 0 ‘***’ 0.001 ‘**’ 0.01 ‘*’ 0.05 ‘.’ 0.1 ‘ ’ 1

S 1.2. PROPORTIONS OF SUBSTITUTED AAS-SAMPLES

SUMMARY
SUBSTITUTED AAS-SAMPLES (Other ingredients then declared):
44% (CI-95: 0.27 T0 0.63) OVERALL; WITH HIGH HETEROGENEITY (92%); SIGNIFICANTLY HIGHER (p = 0.05) IN EUROPE (51%) THAN IN BRAZIL (28%).
Meta-regression by publication year showed a decreasing trend of substituted AAS-samples over time (p < 0.05)
(could be due to Musshoff, 1997 was an outlier)

Review: Substituted

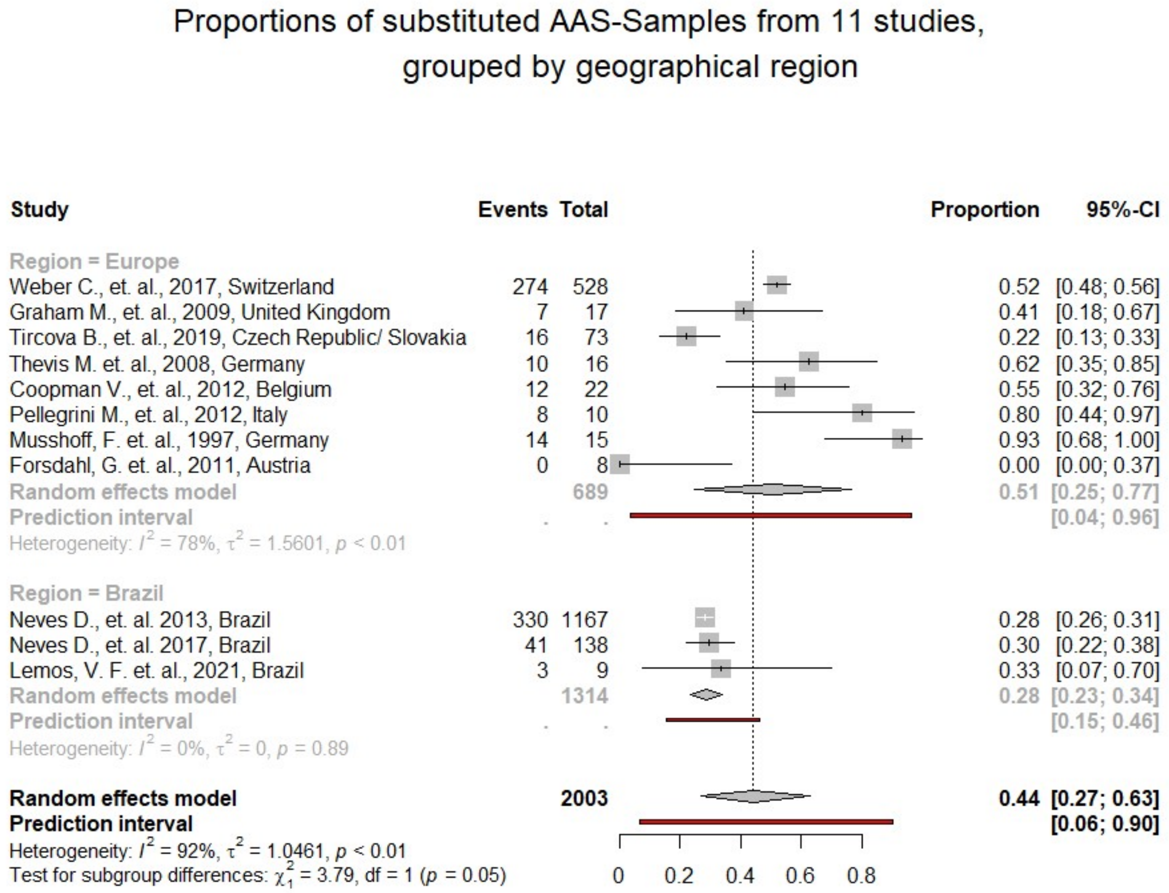

Number of studies combined: k = 11
Number of observations: o = 2003
Number of events: e = 715

	**proportion**	**95%-CI**
Random effects model	0.4419	[0.2692; 0.6300]
Prediction interval		[0.0645; 0.9009]

Quantifying heterogeneity:
tau^2 = 1.0461; tau = 1.0228; I^2 = 91.5% [86.8%; 94.5%]; H = 3.43 [2.75; 4.28]

Test of heterogeneity:
Q d.f. p-value Test

117.75   10 < 0.0001   Wald-type
141.26   10 < 0.0001 Likelihood-Ratio

Results for subgroups (random effects model):

	k	proportion	95%-CI	tau^2	tau	Q	I^2
Region = Europe	8	0.5083	[0.2465; 0.7656]	1.5601	1.2490	32.18	78.2%
Region = Brazil	3	0.2846	[0.2342; 0.3411]	0	0	0.23	0.0%

Test for subgroup differences (random effects model):

	Q	d.f.	p-value
Between groups	3.79	1	0.0516

Prediction intervals for subgroups:

	95%-PI
Region = Europe	[0.0374; 0.9649]
Region = Brazil	[0.1547; 0.4639]

Details on meta-analytical method:
- Random intercept logistic regression model
- Maximum-likelihood estimator for tau^2
- Hartung-Knapp adjustment for random effects model
- Logit transformation
- Clopper-Pearson confidence interval for individual studies
- Continuity correction of 0.5 in studies with zero cell frequencies
 (only used to calculate individual study results)

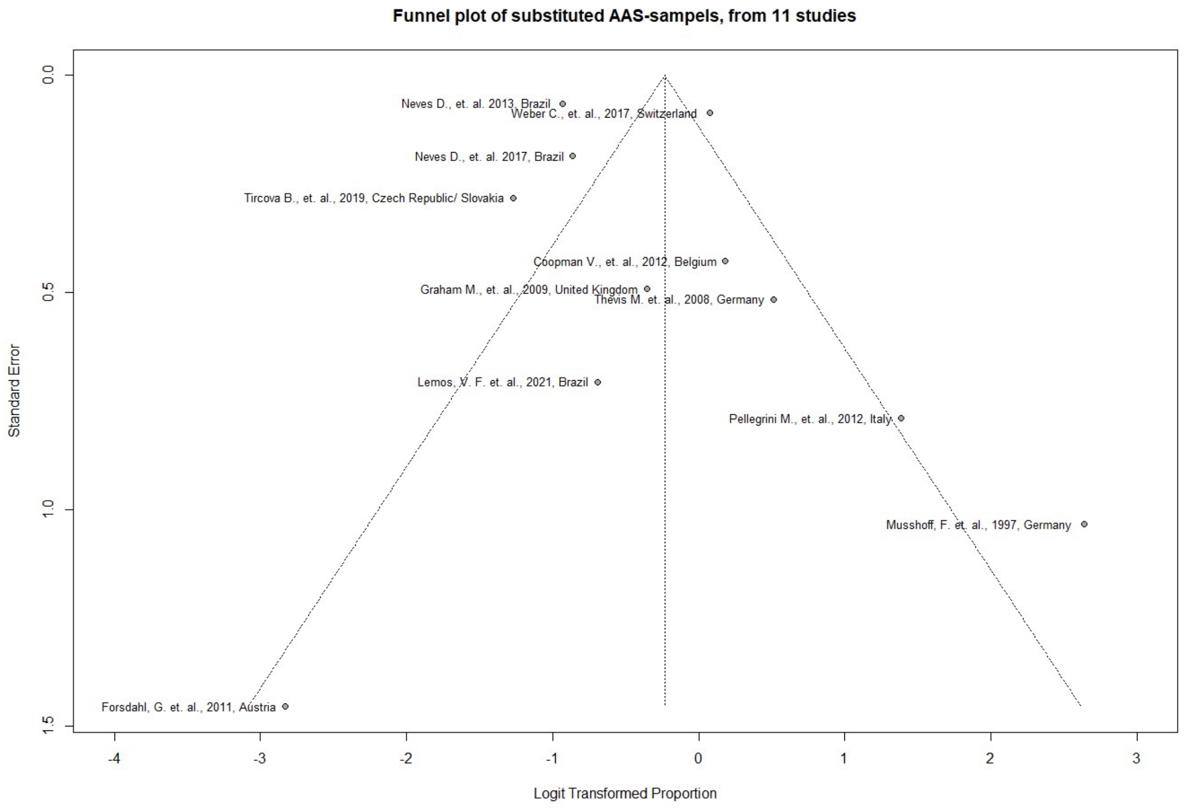

Review: Substituted

Linear regression test of funnel plot asymmetry

Details:
- multiplicative residual heterogeneity variance (tau^2 = 30.5153)
- predictor: inverse of total sample size
- weight: inverse variance of average event probability
- reference: Peters et al. (2006), JAMA

Test result: t = 0.86, df = 9, p-value = 0.4109

Sample estimates:

bias	se.bias	intercept	se.intercept
11.1779	12.9628	-0.6281	0.1875

Meta-Regression by publication year

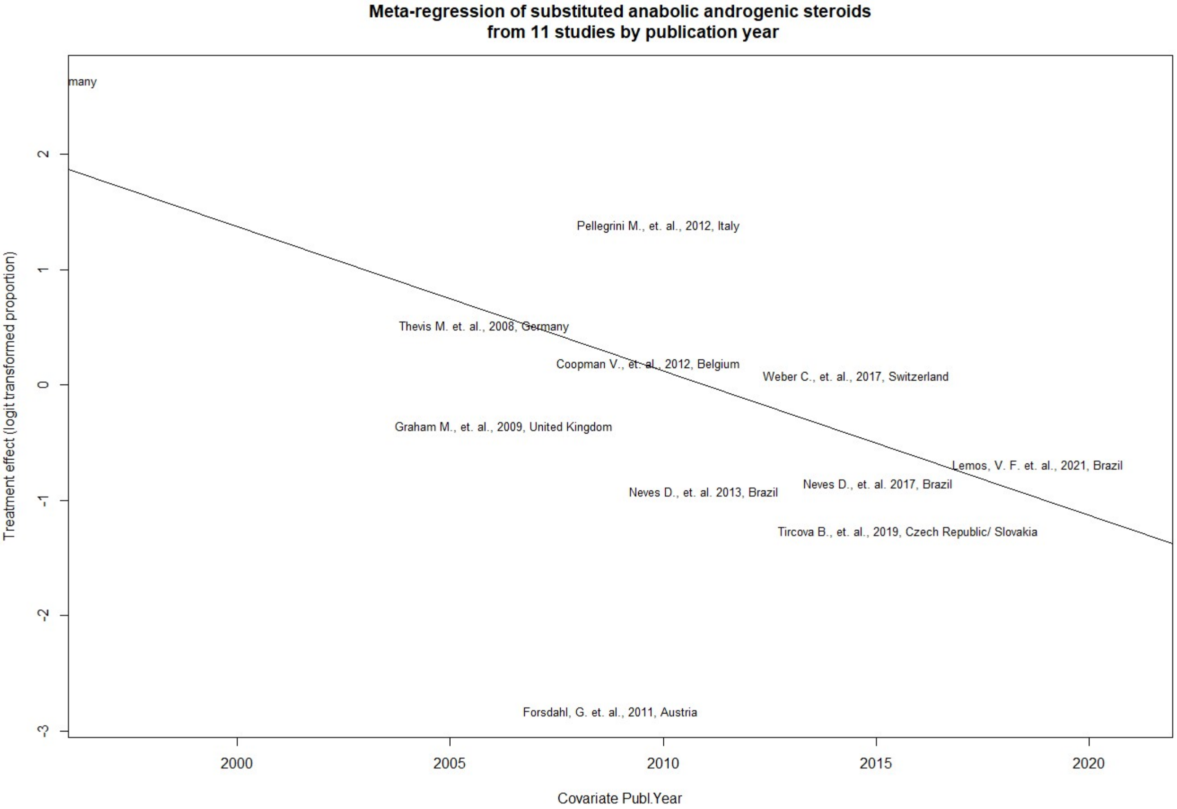

Mixed-Effects Model (k = 11; tau^2 estimator: ML)

tau^2 (estimated amount of residual heterogeneity): 0.3928
tau (square root of estimated tau^2 value): 0.6268
I^2 (residual heterogeneity / unaccounted variability): 87.0071%
H^2 (unaccounted variability / sampling variability): 7.6965

Tests for Residual Heterogeneity:
Wld(df = 9) = 105.0720, p-val < .0001
LRT(df = 9) = 139.3046, p-val < .0001

Test of Moderators (coefficient 2):
F(df1 = 1, df2 = 9) = 8.1312, p-val = 0.0190

Model Results:

	estimate	se	tval	df	pval	ci.lb	ci.ub	
intrcpt	251.5863	88.3322	2.8482	9	0.0191	51.7649	451.4076	*
Publ.Year	-0.1251	0.0439	-2.8515	9	0.0190	-0.2244	-0.0259	*

Signif. codes: 0 ‘***’ 0.001 ‘**’ 0.01 ‘*’ 0.05 ‘.’ 0.1 ‘ ’ 1

S 1.3. PROPORTION OF ADULTERATED AAS-SAMPLES

SUMMARY
ADULTERATED AAS-SAMPLES (= more ingredients then declared):
11% (CI-95: 0.02 T0 0.42) OVERALL; WITH HIGH HETEROGENEITY (92%); NO SIGNIFICANT DIFFERENCE (p = 0.47) BETWEEN EUROPE (13%) AND BRAZIL (5%).
Meta-regression by publication year showed no significant effect.

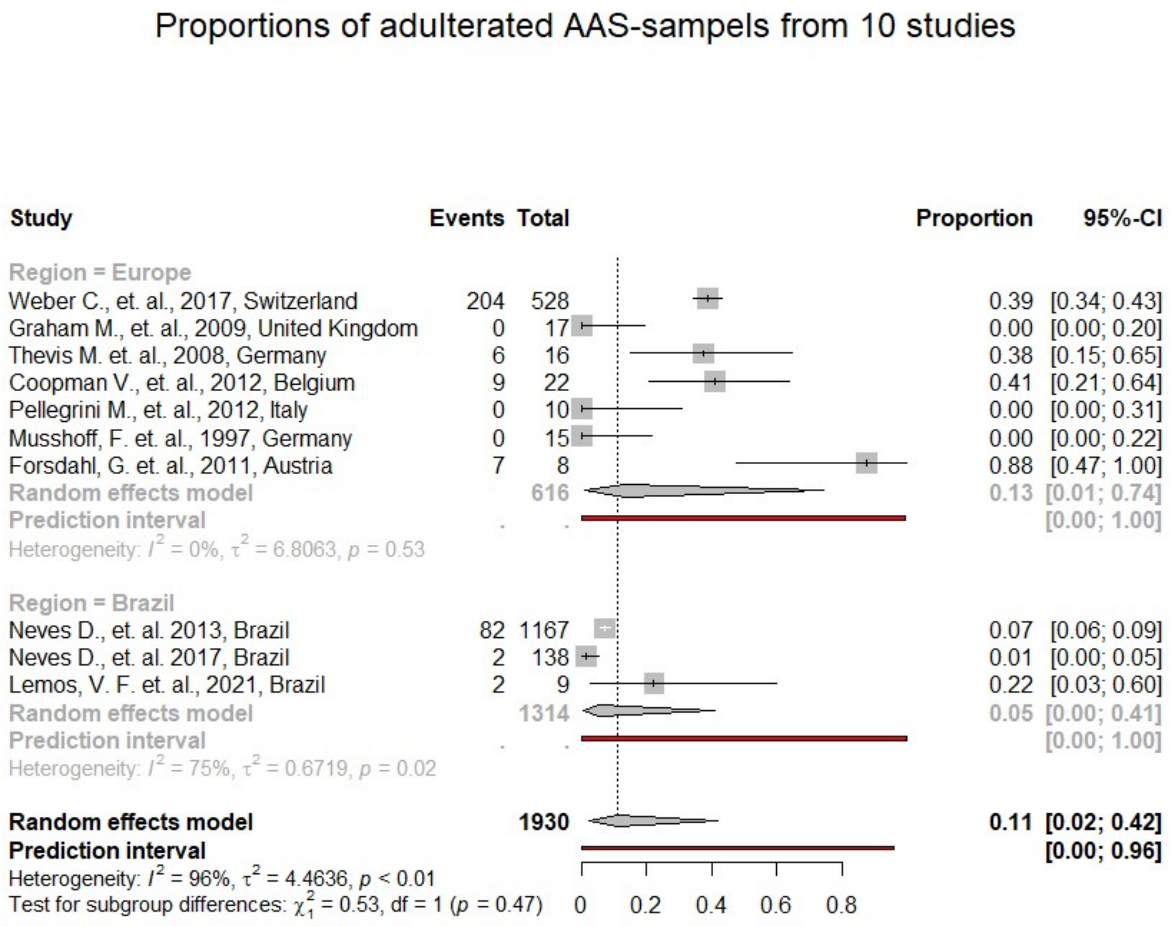

Number of studies combined: k = 10
Number of observations: o = 1930
Number of events: e = 312

	**proportion**	**95%-CI**
Random effects model	0.1125	[0.0221; 0.4162]
Prediction interval		[0.0007; 0.9575]

Quantifying heterogeneity:
tau^2 = 4.4636; tau = 2.1127; I^2 = 96.3% [94.7%; 97.4%]; H = 5.22 [4.36; 6.25]

Test of heterogeneity:

Q	d.f.	p-value	Test
245.49	9	< 0.0001	Wald-type
322.24	9	< 0.0001	Likelihood-Ratio

Results for subgroups (random effects model):

	k	proportion	95%-CI	tau^2	tau	Q	I^2

Region = Europe 7 0.1315 [0.0079; 0.7419] 6.8063 2.6089 5.09 0.0%
Region = Brazil 3 0.0540 [0.0047; 0.4090] 0.6719 0.8197 8.02 75.0%

Test for subgroup differences (random effects model):

	Q	d.f.	p-value
Between groups	0.53	1	0.4653

Prediction intervals for subgroups:

	95%-PI
Region = Europe	[0.0001; 0.9959]
Region = Brazil	[0.0000; 0.9999]

Details on meta-analytical method:
- Random intercept logistic regression model
- Maximum-likelihood estimator for tau^2
- Hartung-Knapp adjustment for random effects model
- Logit transformation
- Clopper-Pearson confidence interval for individual studies
- Continuity correction of 0.5 in studies with zero cell frequencies
  (only used to calculate individual study results)

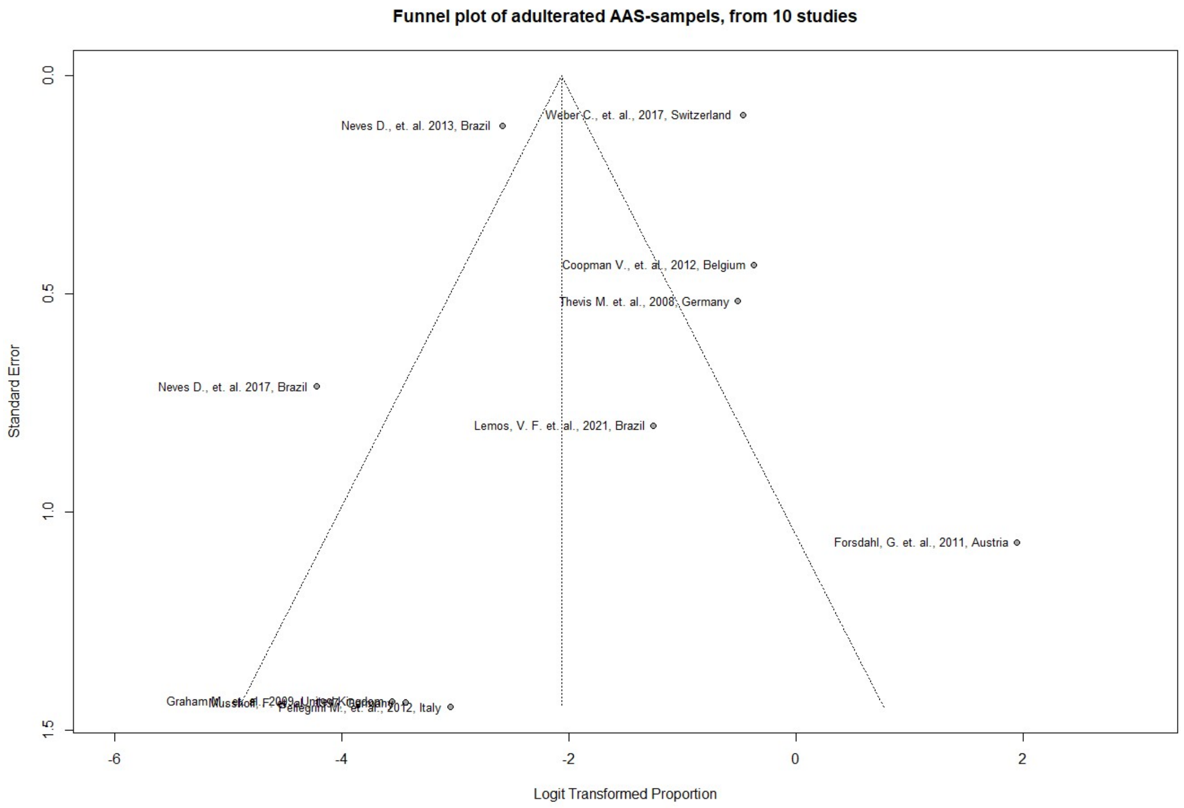

Review: Adulterated

Linear regression test of funnel plot asymmetry

Test result: t = 0.50, df = 8, p-value = 0.6335

Sample estimates:

bias	se.bias	intercept	se.intercept
14.6240	29.5095	-1.3168	0.4895

Details:
- multiplicative residual heterogeneity variance (tau^2 = 46.8001)
- predictor: inverse of total sample size
- weight: inverse variance of average event probability
- reference: Peters et al. (2006), JAMA

Meta-regression by publication year

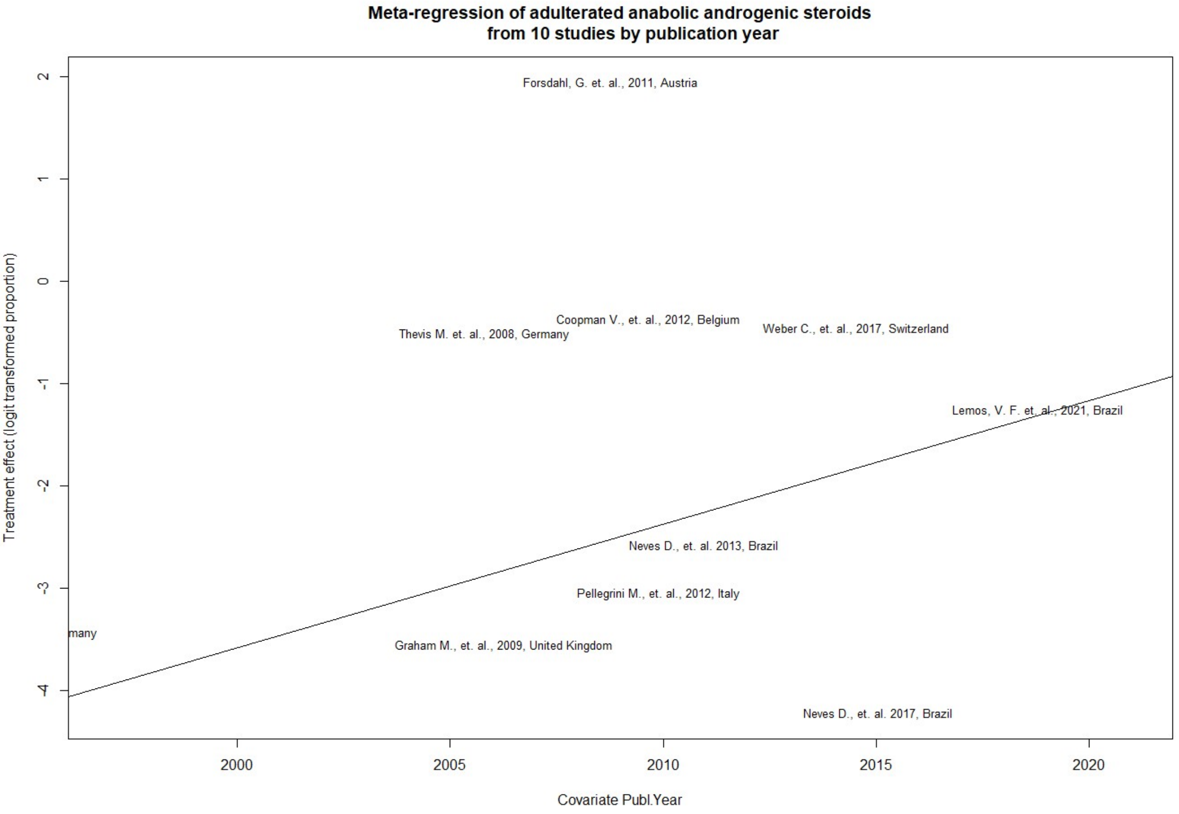

Mixed-Effects Model (k = 10; tau^2 estimator: ML)

tau^2 (estimated amount of residual heterogeneity): 4.5401
tau (square root of estimated tau^2 value): 2.1307
I^2 (residual heterogeneity / unaccounted variability): 96.9090%
H^2 (unaccounted variability / sampling variability): 32.3515

Tests for Residual Heterogeneity:
Wld(df = 8) = 137.7571, p-val < .0001
LRT(df = 8) = 199.2876, p-val < .0001

Test of Moderators (coefficient 2):
F(df1 = 1, df2 = 8) = 0.6845, p-val = 0.4320

Model Results:

	estimate	se	tval	df	pval	ci.lb	ci.ub
intrcpt	-244.8259	293.4994	-0.8342	8	0.4284	-921.6368	431.9850
Publ.Year	0.1206	0.1458	0.8273	8	0.4320	-0.2156	0.4568

S2.1 PROPORTION OF OVER-CONCENTRATED AAS-SAMPLES

SUMMARY

Over-concentrated AAS-SAMPLES (= higher concentration than anticipated):
33% (CI-95: 0.06 T0 0.81) OVERALL; WITH HIGH HETEROGENEITY (96%); SIGNIFICANTLY LOWER (p < 0.01) IN EUROPE (12%) THAN IN BRAZIL (64%).
Meta-regression by publication year showed no significant effect.

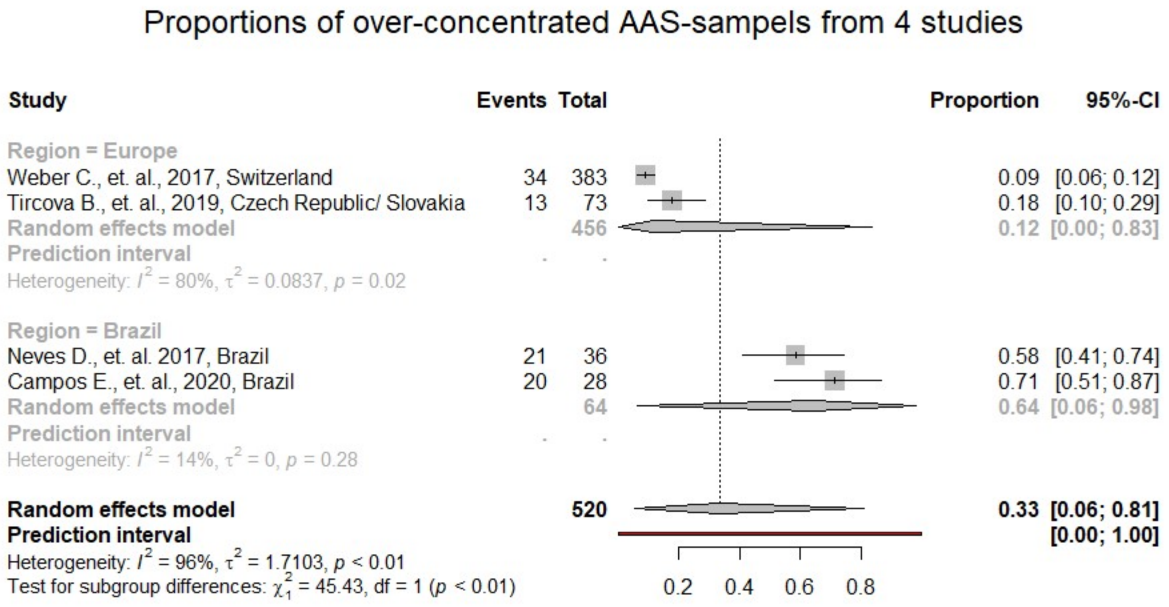

Number of studies combined: k = 4
Number of observations: o = 520
Number of events: e = 88

	proportion	95%-CI
Random effects model	0.3348	[0.0557; 0.8111]
Prediction interval		[0.0009; 0.9965]

Quantifying heterogeneity:

tau^2 = 1.7103; tau = 1.3078; I^2 = 96.4% [93.4%; 98.1%]; H = 5.29 [3.90; 7.17]

Test of heterogeneity:

Q	d.f.	p-value	Test
83.89	3	< 0.0001	Wald-type
92.49	3	< 0.0001	Likelihood-Ratio

Results for subgroups (random effects model):

	k	proportion	95%-CI	tau^2	tau	Q	I^2
Region = Europe	2	0.1155	[0.0034; 0.8339]	0.0837	0.2893	5.08	80.3%
Region = Brazil	2	0.6406	[0.0611; 0.9799]	0	0	1.16	14.0%

Test for subgroup differences (random effects model):

	Q	d.f.	p-value
Between groups	45.43	1	< 0.0001

Details on meta-analytical method:
- Random intercept logistic regression model
- Maximum-likelihood estimator for tau^2
- Hartung-Knapp adjustment for random effects model
- Logit transformation
- Clopper-Pearson confidence interval for individual studies

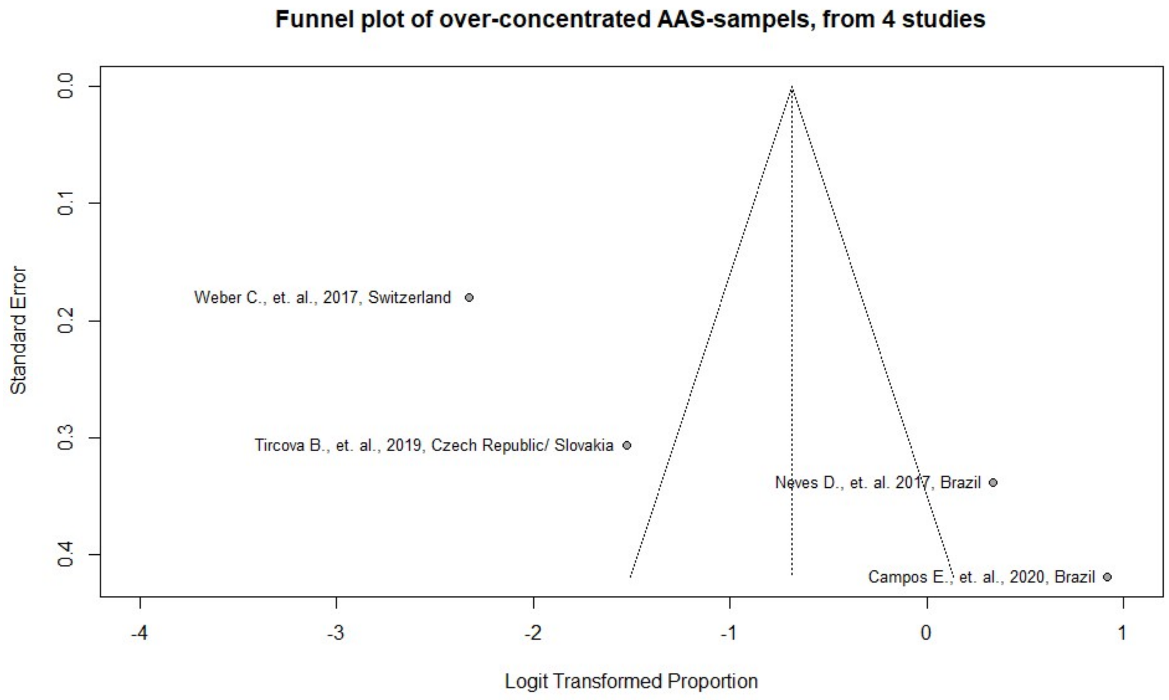

Linear regression test of funnel plot asymmetry

Number of studies (k=4) too small to test for small study effects

Meta-Regression by publication year

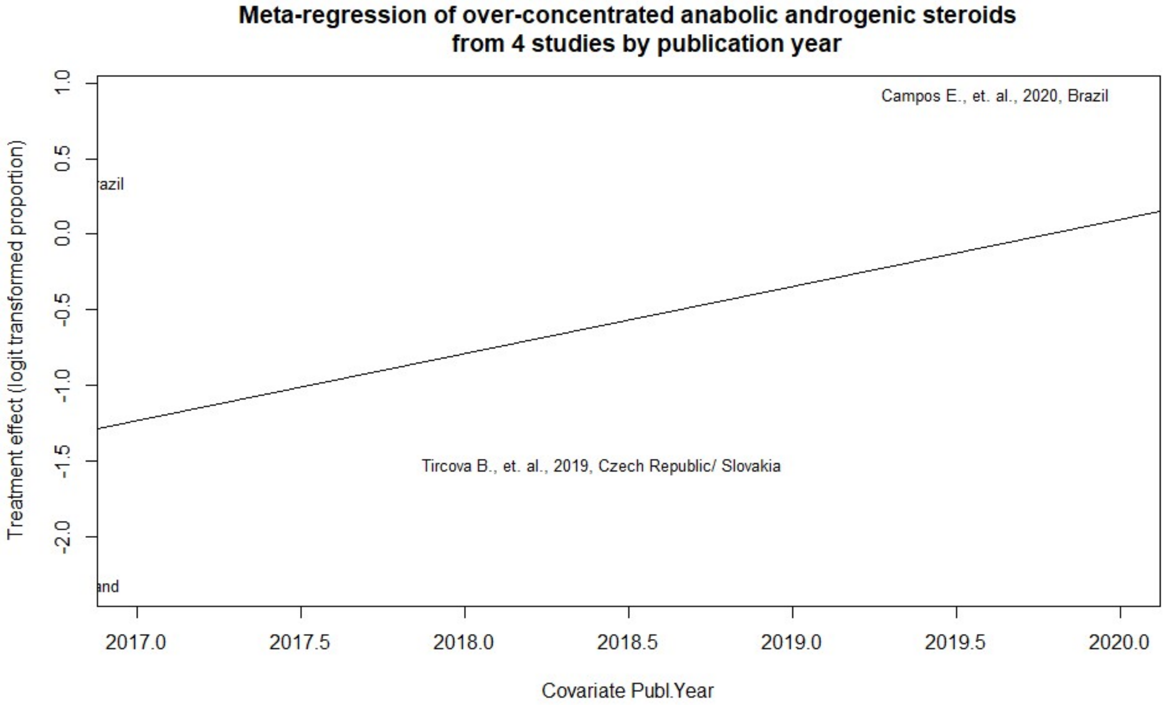

Mixed-Effects Model (k = 4; tau^2 estimator: ML)

tau^2 (estimated amount of residual heterogeneity): 1.3825
tau (square root of estimated tau^2 value): 1.1758
I^2 (residual heterogeneity / unaccounted variability): 93.9229%
H^2 (unaccounted variability / sampling variability): 16.4552

Tests for Residual Heterogeneity:
Wld(df = 2) = 62.4323, p-val < .0001
LRT(df = 2) = 62.3534, p-val < .0001

Test of Moderators (coefficient 2):
F(df1 = 1, df2 = 2) = 0.8862, p-val = 0.4459

Model Results:

	estimate	se	tval	df	pval	ci.lb	ci.ub
intrcpt	-897.0918	952.2405	-0.9421	2	0.4456	-4994.2520	3200.0684
Publ.Year	0.4442	0.4718	0.9414	2	0.4459	-1.5859	2.4742

Signif. codes: 0 ‘***’ 0.001 ‘**’ 0.01 ‘*’ 0.05 ‘.’ 0.1 ‘ ’ 1

S2.2 PROPORTION OF UNDER-CONCENTRATED AAS-SAMPLES

SUMMARY (S2.2 is the inverse of S2.1)

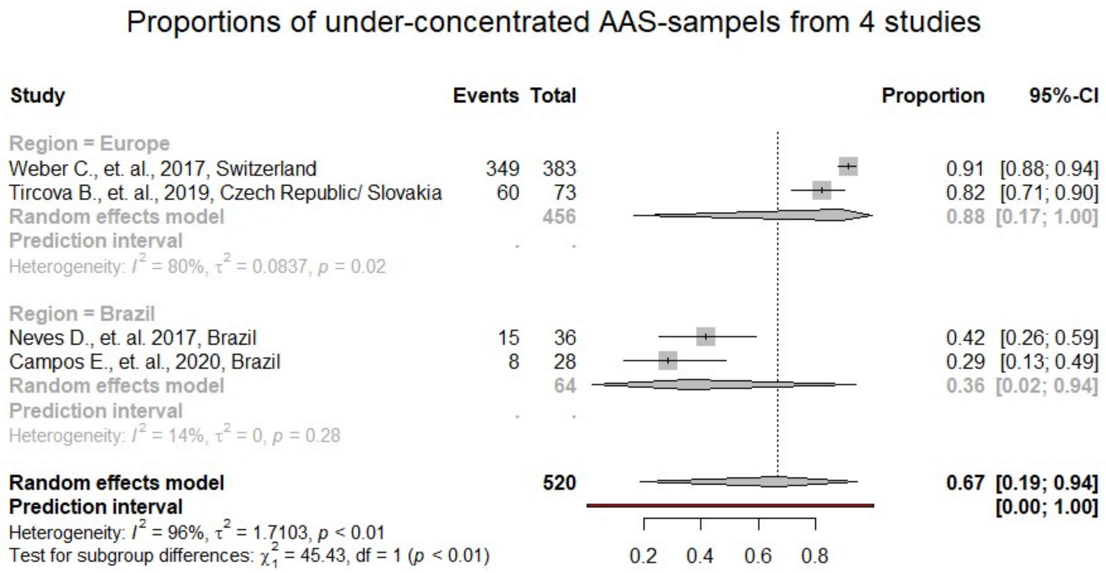

Number of studies combined: k = 4
Number of observations: o = 520
Number of events: e = 432

	**proportion**	**95%-CI**
Random effects model	0.6652	[0.1889; 0.9443]
Prediction interval		[0.0035; 0.9991]

Quantifying heterogeneity:

tau^2 = 1.7103; tau = 1.3078; I^2 = 96.4% [93.4%; 98.1%]; H = 5.29 [3.90; 7.17]

Test of heterogeneity:

Q	d.f.	p-value	Test
83.89	3	< 0.0001	Wald-type
92.49	3	< 0.0001	Likelihood-Ratio

Results for subgroups (random effects model):

	k	proportion	95%-CI	tau^2	tau	Q	I^2
Region = Europe	2	0.8845	[0.1661; 0.9966]	0.0837	0.2893	5.08	80.3%
Region = Brazil	2	0.3594	[0.0201; 0.9389]	0	0	1.16	14.0%

Test for subgroup differences (random effects model):

	Q	d.f.	p-value
Between groups	45.43	1	< 0.0001

Details on meta-analytical method:

- Random intercept logistic regression model

- Maximum-likelihood estimator for tau^2
- Hartung-Knapp adjustment for random effects model
- Logit transformation
- Clopper-Pearson confidence interval for individual studies

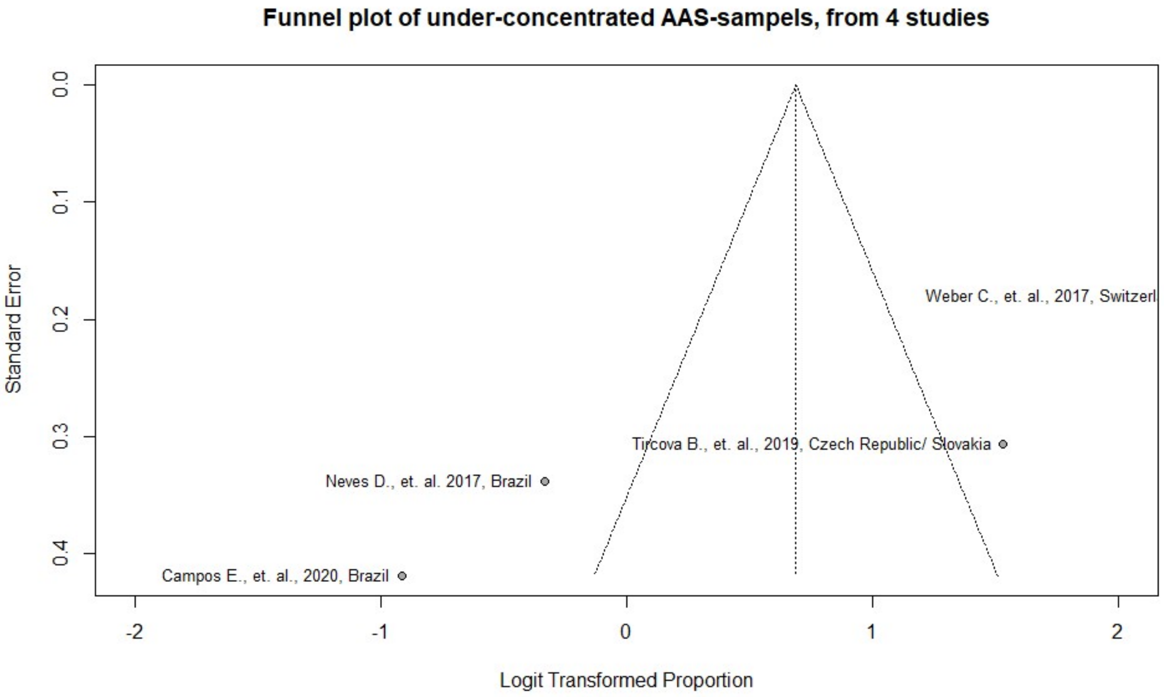

Linear regression test of funnel plot asymmetry
Number of studies (k=4) too small to test for small study effects

Meta-regression by publication year

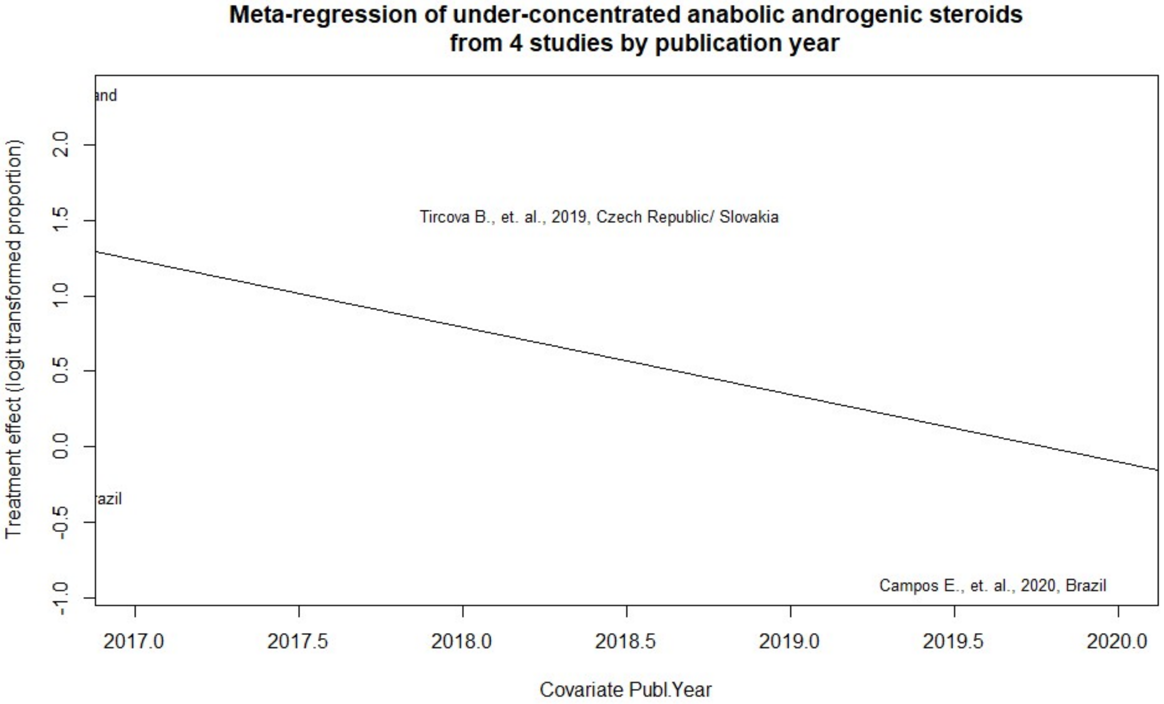

Mixed-Effects Model (k = 4; tau^2 estimator: ML)

tau^2 (estimated amount of residual heterogeneity): 1.3825
tau (square root of estimated tau^2 value): 1.1758
I^2 (residual heterogeneity / unaccounted variability): 93.9229%
H^2 (unaccounted variability / sampling variability): 16.4552

Tests for Residual Heterogeneity:
Wld(df = 2) = 62.4323, p-val < .0001
LRT(df = 2) = 62.3534, p-val < .0001

Test of Moderators (coefficient 2):
F(df1 = 1, df2 = 2) = 0.8862, p-val = 0.4459

Model Results:

	estimate	se	tval	df	pval	ci.lb	ci.ub
intrcpt	897.1058	952.2406	0.9421	2	0.4456	-3200.0548	4994.2663
Publ.Year	-0.4442	0.4718	-0.9414	2	0.4459	-2.4742	1.5859

Signif. codes: 0 ‘***’ 0.001 ‘**’ 0.01 ‘*’ 0.05 ‘.’ 0.1 ‘ ’ 1
